# Supplementary material for: Protocol for the crowdsourced image-based morbidity hotspot surveillance for neglected tropical diseases (CIMS-NTDs)
Source: PLoS One. 2024 May 10;19(5):e0303179. doi: 10.1371/journal.pone.0303179 (PMC11086871; doi:10.1371/journal.pone.0303179)
Supplement: S1 File — (ZIP) [file pone.0303179.s001.zip › NHREC Ethical Approval_Crowdsourced Image-based Morbidity Hotspot Surveillance for Neglected Tropical Diseases in Nigeria.pdf]

National Health Research Ethics Committee of Nigeria (NHREC)

Promoting Highest Ethical and Scientific Standards for Health Research in Nigeria

Federal Ministry of Health

NHREC Protocol Number NHREC/01/01/2007- 28/11/2022
NHREC Approval Number NHREC/01/01/2007-16/01/2023
Date: 16th January, 2023

Re: Crowdsourced Image-based Morbidity Hotspot Surveillance for Neglected Tropical Diseases in Nigeria

Health Research Committee assigned number: NHREC/01/01/2007

Name of Principal Investigator: **Dr. Uchechukwu M. Chukwuocha**

Address of Principal Investigator: Department of Public Health
Federal University of Technology, Owerri
Email: uchukwocha@gmail.com, uche.chukwocha@foto.edu.ng
Tel: +234 8034712957

Date of receipt of valid application: 28/11/2022

Date when final determination of research was made: 16-01-2023

Notice of Expedited Committee Review and Approval

This is to inform you that the research described in the submitted protocol, consent form, advertisement and other participant information materials have been reviewed and *given expedited committee approval by the National Health Research Ethics Committee.*

This approval dates from 16/01/2023 to 15/01/2024. If there is delay in starting the research, please inform the HREC so that the dates of approval can be adjusted accordingly. Note that no participant accrual or activity related to this research may be conducted outside of these dates. *All informed consent forms used in this study must carry the HREC assigned number and duration of HREC approval of the study.* In multiyear research, endeavour to submit your annual report to the HREC early in order to obtain renewal of your approval and avoid disruption of your research.

The National Code for Health Research Ethics requires you to comply with all institutional guidelines, rules and regulations and with the tenets of the Code including ensuring that all adverse events are reported promptly to the HREC. No changes are permitted in the research without prior approval by the HREC except in circumstances outlined in the Code.

The HREC reserves the right to conduct compliance visit to your research site without previous notification.

Signed

Professor Zubairu Iliyasu MBBS (UniMaid), MPH (Glasg.), PhD (Shef.), FWACP, FMCPH, FFPH(UK)
Chairman, National Health Research Ethics Committee of Nigeria (NHREC)
