# Supplementary material for: Protocol for the crowdsourced image-based morbidity hotspot surveillance for neglected tropical diseases (CIMS-NTDs)
Source: PLoS One. 2024 May 10;19(5):e0303179. doi: 10.1371/journal.pone.0303179 (PMC11086871; doi:10.1371/journal.pone.0303179)

Crowdsourced Image-Based Morbidity Hotspot Surveillance for Neglected Tropical Diseases PROJECT PROTOCOL

Innovations and Technologies for Disease Control (INTEDeC) Research Group, Department of Public Health, Federal University of Technology, Owerri, Nigeria.

Section	Item	Procedure/ Description
Project Overview	Project Title	Crowdsourced Image-based Morbidity Hotspot Surveillance for Neglected Tropical Diseases in Nigeria
	Protocol summary	This protocol involves the methods and procedures for the execution of the research project on crowdsourced image-based Morbidity Hotspot Surveillance for Neglected Tropical Diseases in Nigeria. The project involves three parts; 1)Sensitization of NTDs affected subjects or those around them in their communities to take photographs of the NTDs manifestations upon notice with a smart device and transmit together with relevant demographics to a designated telephone number through social media handles, from where they are collated in an electronic data base, identified, and matched to specific NTDs by domainexperts. This, together with location data will be used to map the specific NTDs as well as to determine their demographic and geographic distributions. 2) With the data base of telephone numbers collated through the transmission of photos, subjects will be personally visited and given feedbacks on preliminary diagnosis of their conditions and appropriate immediate actions to take for morbidity management and disability prevention, supportive non-clinical care such as counselling on hygiene practices and avoidance of transmission enabling behaviors. 3) Large volume country wide collection of images of NTDs manifestations to generate dataset to be used for the development and training of an Artificial Intelligence (AI)/ Machine Learning (ML)algorithm for eventual identification and matching of received images to specific NTDs as well as preparatory to the development of a point of care AI-driven mobile application for differential diagnosis and management of NTDs at health facility levels.

	Investigators	• Principal Investigator<br>Dr UchechukwuChukwuocha<br><br>• Co-Investigators<br>Dr Obiageli Nebe<br>Mr Emmanuel Davies<br>Dr Ikechukwu Ayogu<br>Dr Godswill Ekeleme<br>Mr Greg Iwuoha
	Collaborators	• Collaborators/ Partners<br>- National programme for the elimination of NTD, Federal Ministry of Health (FMH), Nigeria<br>- Visual Dx,Five Nigerian NTD experts of over 20 years' experience in NTDs field research.
	Funding	• Funding<br>Bill and Melinda Gates Foundation
Introduction	Background	Neglected Tropical Diseases (NTDs) have been classified with goals of elimination by the World Health Organization (WHO) in the NTDs Road Map 2021-2030 [1]. Recently (in 2022), the Kigali declaration on NTDs was made to mobilize political will and secure commitments to achieve the Sustainable Development Goal 3 (SDG3: Good Health and Well-being) target on NTDs and to deliver the targets set out in the World Health Organization's Neglected Tropical Disease Roadmap[2].Effective surveillance and targeted deployment of interventions are essential components of strategies towards the elimination of NTDs. Traditional surveillance systems are however time-consuming, expensive, and do not cover the entire population. Additionally, they are ineffective in conditions that prevent access to locations where NTDs may be endemic, such as the case of the COVID-19 pandemic, civil upheavals, and natural catastrophes. There is therefore need to develop novel NTDs surveillance methods that will overcome the challenges of the traditional methods and effectively aid the actualization of the NTDs elimination agenda. Surveillance based on crowdsourced data has been shown to provide an affordable, ~~realtime~~real time, scalable and effective method

		to improve malaria surveillance in the recent past [3]. This project therefore seeks to contribute towards achieving the WHO target for the elimination of NTDs by developing a crowdsourced image-based morbidity surveillance method to improve the surveillance of NTDs.
	Justification for study	Traditional surveillance systems of NTDs are time-consuming, expensive, and often times not population inclusive, giving rise to missed cases. They may not be effective for post elimination surveillance as well. Additionally, they are ineffective in conditions that prevent access to locations where NTDs may be endemic, such as the case of the COVID-19 pandemic, civil upheavals, and natural catastrophes.
	Intended/Potential use of study findings	• To provide data for better planning and targeted deployment of interventions for elimination of NTDs<br>• For more effective NTDs morbidity management and disability prevention<br>• To provide an effective tool for NTDs post elimination surveillance<br>• To reduce cost of NTDs surveillance<br>• To expand equitable access to accurate diagnosis, referral, and care for NTDs patients<br>• To prepare for possible full automation of NTDs surveillance and response using AI/ML algorithms and crowdsourcing tools<br>• To prepare towards integration of this method into on-going plans and programs for NTDs elimination and into the health system in general
	Study design/locations	• Implementation research<br>• Study location is Nigeria<br>Three implementation States and three control States.

	Objectives	• To develop and validate an electronically transmitted crowdsourced photograph/ image (remote) method to improve the surveillance of NTDs<br>• To develop and validate a feedback mechanism and personalized/individual care for NTDs subjects<br>• To develop and train an Artificial Intelligence (AI)/ Machine Learning (ML) algorithm for eventual identification and matching of received images to specific NTDs as well as preparatory to the development of a point of care Artificial Intelligence (AI) driven mobile application for differential diagnosis and management of NTDs at health facility levels
Procedures/ Methods Design	General approach	To develop a crowdsourced image-based morbidity surveillance method to improve the surveillance of NTDs
	Ethical clearance/considerations	• Obtain ethical clearance from relevant bodies<br>- National Health Research Ethics Committee of Nigeria (NHREC)<br>- Institutional Review Board of Federal University of Technology, Owerri, Nigeria<br><br>• Ethical Considerations<br>-Advertisements/notice to bear a brief description of the project, its intentions, and the role of potential participants in lay language, and request that only those who give consent to participate should transmit images of NTDs manifestations (without their faces) and accompanying demographics (without their names).<br>- Automated response from the receiving WhatsApp or Telegram platforms prompting those who transmit images/ data to confirm their consent and permission to use their data in the research as a means of validating consent<br>- Community contact persons who will assist those without smart devices to take photographs of NTDs manifestation and transmit should collect informed written consent from such subjects<br>- Hard copies of signed consent formsto be

		collected from community contact persons, digitized and stored in a well-secured electronic archive, before permanent storage in a secure steel cabinet.Electronic consent shouldbe stored in well-secured electronic database.<br>- For children and vulnerable groups, their parents and care giversare to provide consent on their behalf with the children assenting to it.
	Privacy and Confidentiality	• While collecting and transmitting images, in case of appearance of body parts apart from the image of the manifestation of NTDs, such a body part should be cropped out<br>• Redact picture other than the NTDs manifestation to strip all individual identifiers<br>• Create restriction for data sharing including, pictures, using computer generated passwords or a two-factor authentication from Google that is only accessible by the Principal Investigator and Program Manager<br>• Adopt strategies to minimize risk of disclosing subjects' identities such as throughanonymization of pictures using codes to prevent identification of subjects, statistically altering the data in ways that should not compromise secondary analyses.<br>• Personnel or trusted parties who may be granted privileged, direct access to the data and pictures shall commit to protect privacy and confidentiality of the data. Access to data shall be provided through a controlled site like an encrypted cloud storage system<br>• In case of institutional or other group request for data or images, hybrid methods, releasing redacted dataset for general use but providing access to more sensitive data through a user contract or data enclave.<br>• Prevent risk of misinterpretation of data/ picture using annotated codebooks and data documentation.<br>• All data users shouldbe bound by the

		principles of data access that specify good stewardship of data<br>• Responsibility for appropriate use of data shouldlie with the investigators and personnel that have access to the dataImagesshould not be shared on any social media platform<br>• Obtain a written consent from every subject that will be transferring photograph through a third party.<br>• During advertisement and publicity, ensure that information regarding picture sharingconspicuously providedclearly stated that by sharing their picture they are providing consent.
	Audience and stakeholder participation	• There is need to create strategy partnership with stakeholders as follows:<br>- Build partnership with Federal Ministry of Health National NTDs elimination programme who will, upon request, supply information particularly on endemicity of NTDs in the country, validate photographs and message, approve protocol and issue ethical clearance, introduce project team to State and Local Government NTDs elimination coordinating offices, create a triage between state, local for the elimination of NTDs for effective management of identified cases and deployment of interventions<br>• Create strategic partnerships with non-governmental organizations working in the project implementation areas who will support with community sensitization/ mobilization<br>• Synergize with local/community stakeholders in NTDs implementation communities who will equally support community sensitization/ mobilization
	Cost benefit	To provide a real-time, inexpensive, population inclusive, feasible, acceptable and more effective method as compared to the traditional/ conventional method of surveillance of NTDs
	Study timeline	18 months
Study location/		Study location is Nigeria. Nigeria is located

Population		in West Africa, and between latitudes 4 and 14 degrees north and 2 and 15 degrees east, sharing a border with Benin, Niger, Chad and Cameroon. The country has a population of over 216.7 million people with a population density of 226 per Km^2. Nigeria has 36 states which are grouped into 6 geopolitical zones which are North-East, South-East, South-South, South-West, North-West and North-Central. It has a range of natural environments, from semi-deserts in the north to tropical rainforest in the south. Nigeria has a tropical climate with variable rainy and dry seasons, depending on location. It is hot and wet most of the year in the southeast but dries in the southwest and farther inland. Nigeria is one of the most densely populated nations in Africa, the most populous country in the African continent, and the seventh-most populous nation in the world. Hence, Nigeria is a likely target market for mobile phone, internet, and telecommunication businesses, and as such has influenced many Nigerians to becoming smart phones owners. It is estimated that about 90% of the country's population are mobile phone owners [4]. Nigeria has a significant amount of cultural diversity, due to its more than 250 distinct ethnic groups, more than 500 languages, and the variety of traditions practiced by each of them. Nigeria's economic situation is such that more than 40% of the population lives in poverty, with poor social and healthcare infrastructure. This situation together with environmental and climatic factors encourages the spread of a variety of diseases, including neglected tropical diseases. Nigeria bears the second highest global burden of NTDs.
	Case definitions	Manifestations of NTDs (NTDs of focus areOnchocerciasis, Lymphatic filariasis, Trachoma, Buruli Ulcer, Guinea worm and Ascariasis)
	Study setting	• Purposively select six states based on data on NTDs prevalence from the Federal Ministry of Health NTDs elimination programme office<br>• Selection of states to cover the geopolitical zones of the country<br>• Randomly select three of the states as

		implementation states and the rest of the three states as control states<br>• Additional criteria for selection of study setting should be the presence and operation of partner NGOs in such states
Intervention		
	Study instruments,	• Smart devices (Mobile Phones, Tablets)- to photograph images of NTDs manifestation and transmit along with demographic data to the receiving electronic storage system.<br>• Structured pre-tested questionnaire- to collect data on subjects' experience, acceptability, usability,and satisfactions with the new method<br>• Semi-structured interview guide – to collect data from State and Local GovernmentNTDs surveillance officers on their experiences with the conventional NTDs surveillance methods and notification officers in the state and their perceptions about the new surveillance system
	Project Implementation	**Month 1**<br>1. **Inception meeting of project partners (Virtual and/or in-person of all project partners).**<br><br>Purpose<br>• Further familiarization of project partners<br>• Assessment of the project design, procedures, and protocols<br>• Assignment of roles<br>• Set up of workflow/system for the project<br><br>2. **Recruitment and training of research assistants**<br>• **S**elect research assistants as post graduate students<br>• Train RAs in data collection and community sensitization/ mobilization<br><br>**Month 2**<br>**1. Design of project advertisement/notice**<br>• Conduct photo and literature search to select photos of manifestations of NTDs of focus (onchocerciasis, Lymphatic filariasis, Trachoma, Buruli Ulcer, Guinea worm and Ascariasis)

		• FMH NPO officials/experts to validate photos using WHO standard operating procedures<br>• Use validated photos to design the project advertisement/notice with content as follows:<br>1)Distinctive photos of the manifestations of the focus NTDs with descriptions in English and local languages of the study locations<br>2) Call for individuals with NTDs manifestations or those who have encountered them to take clean photographs of the manifestation (with the consent of the subject and excluding their faces) using a smart phone, and transmit through designated social media channels (e.g. WhatsApp and Telegram) to phone numbers indicated on the advert, accompanied with demographics of the subject (location, age, gender, occupation, marital status, educational level), or to use a designated community contact person whose name and phone number are on the advert to do so on their behalf in case of lack of a device, capacityor know-howto carry out the activity<br>3) Notice that the NPO would provide available intervention measures to those who comply with the request.<br>• Advert narratives should be in both English language and the respective local languages of the pilot locations.<br><br>**2. Selection of Project Sites/Locations**<br>• Select 6 pilot states purposively based on available data from the Federal Ministry of Health, Nigeria on the endemicity of the target NTDs which covers the six geopolitical regions of the country<br>• Of the six states, select three states randomly as the intervention states while the other three states will serve as controls<br>• States should be selected in endemic states where the Partner NGOs are already operating

		**3. Development of a cloud-based Image Collection and Labelling Software**<br>• Define requirements for the software system<br>• Design and implement the software, capturing the requirements<br>• Evaluate the software and iterate until satisfied<br>• Launch the software and train personnel on its use<br><br>**Month 3**<br>**Advocacy visit and engagement of local stakeholders**<br>• Together with the respective State ministries of health NTDs NPOs, identify local governments/ communities where project will be implemented in the respective states<br>• Pay advocacy visits to such local government/communities<br>• Identify the local stakeholders in such local government/communities such as traditional rulers, community health workers (CHW), patent medicines vendors, native healers,etc.<br>• Organize a workshop for the stakeholders to introduce the project to them, explain the project purpose, procedure and outcomes and their roles in the project as well as solicit their support<br>• Select community contact persons from the respective study communities preferably among community health workers or community directed distributors of ivermectin<br>• Train Community contact persons to use smart devices to capture images of NTDs manifestations, collect demographic data and transmit through WhatsAppto a designated phone number. Training should also be on adhering to privacy and confidentiality as well as obtaining written informed consent as well as follow up of subjects to ensure adherence to recommended interventions

		**Month 4**<br>**1. Intervention communities' sensitization and mobilization**<br>• Together with partner NGOs and community stakeholders, sensitize and mobilize selected intervention communities after stakeholder engagement.<br>• The aim is to create awareness about the project among community members and carrying them along<br>• These should be complemented with some on-going engagement throughout the project to foster community ownership, and allow adaptation for optimization of project implementation<br>• There shouldbe periodic advocacy visits to the project communities and continuous engagement with the community stakeholders physically and virtually to carry them along all through the project in order to foster community ownership.<br><br>**2. *Production and Deployment of Project Advertisements/Notices to Intervention States/ Sites***<br><br>• Produce and deploy validated advertisements/notices to intervention states as follows:<br><br>- Billboards – tobe deployed at strategic locations such as community centers<br>- Posters – tobe posted at primary, secondary and tertiary health facilities, commuter parks, religious centers, markets and schools<br>- Handbills –to be distributed at market squares and other public places.<br>Additional adverts/notices shall be placed on local TV, Radio stations as well as onsocial andin print media<br>• These are aimed at adequately sensitizing the public on the procedures and their involvement in the process<br>• Control states will continue with the traditional surveillance systems without the intervention.

		**Month 4-7**<br>**Collection of large volume of Photographs of Manifestations of NTDs and Development of Artificial Intelligence (AI) driven Matcher of Images to Specific NTDs**<br><br>• The expert consultants to lead the countrywide collection of large volume of images of NTDs manifestation from university researchers, clinicians and other identified experts in Nigeria from their various image collections resulting from their many years of practices. Visualdxshall facilitate the collection of images from other parts of the world<br>• Each expert to independently identify and match each of the received images blindly on the same images. A preliminary diagnosis shall be deemed established when three of the five human annotators concur. This should also serve as a means of quality check among the experts<br>• Generated datasetto be used to train the Artificial Intelligence (AI)/ Machine Learning (ML) algorithm for subsequent automated identification and matching of electronically transmitted crowdsourced photographs of manifestations to specific NTD
		**Month 7-15**<br>**Reception, collation, and processing of photo, demographic, and other data**<br>• After community sensitization and deployment of advert notices, electronic transmission of photo and demographic data from intervention communities should be expected.<br>• Collectand/collate transmitted photos and demographic data in a central electronic database protected with an

		access control mechanism.<br>• Process, clean and sort images based onpredetermined quality parameters such as clarity, replications, and completeness of and completeness of requiredmetadata.<br>• Use internet protocol (IP) of the devices used in transmitting of the photos to check and eliminate repeated submissions<br>• Crop out any body partsnot affected by the NTD, but which is exposed in transmitted images<br>• Anonymize all received data<br>• Organize and set up the image database for annotation by human expert consultants.<br>• Each expert consultant to independently/blindly identify and match each of the received images to specific NTDs, using a guideline developed according to WHO's description of the NTDs manifestations (See table 1). Three of the expertsmust agree in order for a preliminary diagnosis to be established.<br>• Assess and analyzerecords of the ministry of health of both the implementation and control states for their weekly and monthly NTDs surveillance data<br>• Towards the end of the project,<br>1.Use structured, pretested questionnaire to collect data from participants to determine experience, acceptability, usability, and level ofsatisfaction with the new method<br>2.Use semi-structured interview – to collect data from state and local NTDs surveillance and notification officers on experience of the conventional surveillance method and their perception of the new surveillance system
		**Month 7-15**<br>**1.Feedback on Preliminary Diagnosis and immediate actions to take and follow up**<br>• Project Case management officer together with the community contact

		persons to physically provide preliminary diagnosis on the identified NTDs and counselling to the subjects and follow up through phone calls<br>• Provide information on immediate recommended actions to take for morbidity management and disability prevention<br>• Provide counseling to dispel misinformation, misconceptions, and myths as well as on hygiene practices and avoidance of other enabling behaviors<br>• Create a triage (Federal, State, Local Government NTD control/elimination programme offices) for adequate response to cases of NTDs leveraging on existing NTDs control/elimination structures. This includes frontline health facilities in Local Government Areas where identified cases of NTDs are managed and counseled, and referral to tertiary health facilities where more serious cases beyond the capacity of the frontline facilities done<br><br>***2. Data Analysis***<br>• Generate spatial distribution maps of respective focus NTDs in the implementation states using arch Map, or any other digital mapping software<br>• Generate descriptive data on the distribution of focus NTDs in the implementation states represented in tables and figures<br>• Compare the performance of conventional surveillance method (data from weekly and monthly surveillance records) in both the control and the implementation states with the new surveillance method (crowdsourced image-data with accompanying demographics) using the following indicators<br>- Proportion of suspected NTDs cases (disease) per proportion of each of the following cases reported weekly and

		monthly<br>- Rate/timeliness of reporting of suspected cases<br>- Proportion of identified cases that receives some response in the intervention cases compare to the conventional cases<br>- Promptness of the response received, demographic and geographic distribution of identified cases per focus NTDs<br>• Use statistical software packages such as SPSSand Rto analyze quantitative data using T- test<br>• Use NVivo to analyze qualitative data following a general inductive approach
		**Month 7-15**<br>**Development and Training of the Artificial Intelligence/ Machine Learning Platform for the Identification and Matching of Transmitted Images to Specific NTDs**<br><br>***To be led by Visual DX using the following procedure**<br>• AI/ML training and iterative development to support development of an NTD targeted module by VisualDx working in collaboration with Nigeria partners using the data set generated from the largescale collection of images of NTDs manifestation from Nigeria and other part of the world. This is to train a deep neural network for predicting select NTDs including Dracunculiasis, Buruli Ulcer, Visceral leishmaniasis and PKDL, Leprosy, Lymphatic filariasis and Onchocerciasis along with their clinical mimics<br>• Source NTD images from Nigeria through in-country consultants to lead image collection and curation along with other Nigerian NTD experts, faculty members of universities and clinicians who are members of the Parasitology and Public Health Society of Nigeria<br>• Source clinical images from open

		datasets, solicited images and cases from clinicians and international professional societies<br>• Source images for other diagnoses, clinical mimics of the selected NTDs from VisualDx proprietary database of 100,000's of clinical imagesPair down the full list from Visualdx to those pertinent to the differential diagnoses of the selected NTDs<br>• Create clinical questionnaires, working with a panel of experts in collaboration with Nigerian colleagues to incorporate VisualDx NTD module that will collect information about signs and symptoms pertinent to NTD presentations as well as to their clinical mimics<br>• Conduct series of tests and iterative enhancements for each included NTD to demonstrate accuracy and the utility of model (internal validation).<br>• Enable collection of demographic and location data associated with instances of positive NTD syndrome identification to enable public health syndromic surveillance (tracking cases over time, region, and social determinants of health) in the mobile app

	Output	• A curated list of other image-based crowdsourced surveillance in Africa and similar settings.<br>• Protocol for remote surveillance of NTDs through electronic transmission of crowdsourced photographs of NTDs manifestations.<br>• A list of messages advertising the system (promoting the benefits of submitting photos and what will be done with them) validated by the FMOH's national NTD program.<br>• Image dataset of different stages of NTDs manifestations.<br>• Demographic and Geographic distribution data of NTDs distribution.<br>• Data on images of NTDs manifestations physically matched to specific NTDs.<br>• Data from the subjects on their experience, acceptability, usability and feasibility of the new crowdsourced image-based NTDs surveillance method.<br>• Data from local NTDs surveillance officers on their experiences with the conventional surveillance methods and possible perceptions about the new method.<br>• Final Project Report - 30 April 2024.
Monitoring and Evaluation		• Perform checks and balances through monthly project implementation meetings<br>• Monthly project implementation meetings to assess the extent of achievement of project objectives and adherence to timeline<br>• Appoint Monitoring and evaluation officer from the project team to ensure set objectives are achieved within the stipulated project and timeline<br>• Ensure quality control and assurance of all transmitted images by double checking for clarity, replication and complete data and that internet protocol of devices are used to check repeated transmissions<br>• Involve a data protection expert to ensure compliance to relevant data protection laws

		• Expert consultants to work independently to identify and match each of the collected NTDs manifestation images from the largescale image collection process<br>• Initiate Initial metrics to assess accuracy of the AI/ML algorithm involving iterative evaluation against a test set of previously unseen imageswhich will include images not only of the select NTDs, but of their clinical mimics as well as other common acute skin complaints<br>• Use the outcome of the NTDs image identification and matching of NTD images by the expert consultants (Standard) to compare the outcome of the AI/ML process
Methods ofDissemination, Notification, and Reporting of Results		• Conduct seminars at the end of the project to inform all stakeholders of the outcome and implications of the project<br>• Submit the project report to the Bill and Melinda Gates Foundation, the Federal Ministry of Health of Nigeria, as well as State and Local Ministries of health and other local stakeholders.<br>• Present findings at both international and local conferences<br>• Publish findings in peer review journals
Mitigation of potential risk of stigmatization of subjects		• Anonymization of images and demographic data of participants and subjects<br>• Assurance of privacy and confidentiality of data<br>• Continuous community sensitization and awareness on NTDs with generic message in partnership with community stakeholders to reduce misinformation, misconception which are the drivers of stigmatization<br>• Sensitization to also highlight the negative implication stigmatization to the community and its members<br>• Creation of peer support groups in the community using Community group where subjects discuss and encourage themselves to be involved in disease

		control activities and other community development activities<br>• Creation of dedicated help lines through which concerned subjects can reach case management to counsel and address any of their concern
References		1. World Health Organization. Neglected Tropical Diseases, 2021. (accessed 29 November, 2021); Available online: https://www.who.int/neglected _diseases/diseases/en/<br><br>2. Uniting to Combact Neglected Tropical Diseases Coalition. The Kigali Declaration, 2022. (accessed 11 June 2022); Available online: https://unitingtocombatntds.org/kigali-declaration/<br><br>3. Adekembi, O., Oyewusi, W.F., &Ogundepo, E. Rea-Time Crowdsourcing of Health Data in a Low-Income Country: A case Study of Human of Human Data Supply on Malaria First-Line Treatment Policy Tracking in Nigeria. In CWS@Neurips; 2020: 14-18<br><br>4. Gilbert P. Connect Africa: Nigerian Internet and Mobile Penetration Grows, 2021. (accessed 19 August 2022); Available online: https://www.connectafrica.com/author.asp

Table 1: GUIDELINES FOR IDENTIFICATION AND MATCHING OF IMAGES OF NTDs MANIFESTATIONS BY EXPERTS

DISEASE	MOST AFFECTED BODY PARTS	DESCRIPTION OF MANIFESTATION	Images
Buruli ulcer	Typically, limbs (lower and upper limbs)	• Large area of induration<br>• Diffuse swelling of the legs, arms or face (oedema)<br>• Tropical phagedenic ulcers of the legs, with undermined edges and granulating base covered by a yellowish membrane	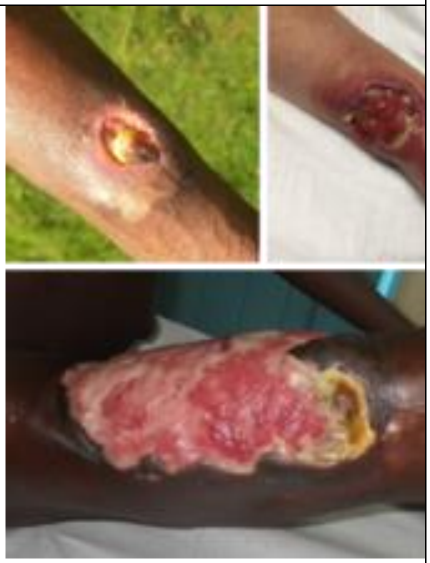
Leprosy	the skin, the peripheral nerves, mucosa of the upper respiratory tract, and the eyes.	• Skin lesions of hypopigmented macules (flat, pale areas of skin), and eye damage (dryness, reduced blinking).<br>• Large ulcerations, loss of digits, skin nodules, and facial disfigurement.<br>• Z-thumbs, clawing, contractures and shortening of fingers due to repetitive injury and healing.<br>• Chronic nonhealing ulcer at the metatarsal head	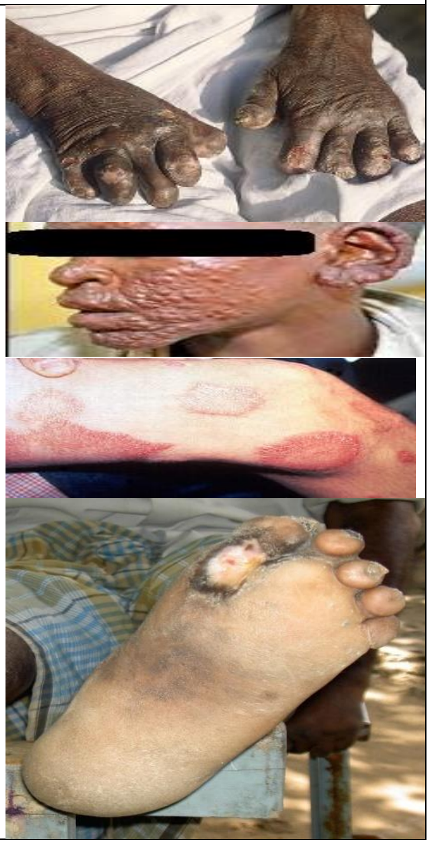

Guinea worm disease	Lower limbs, feet	• A fluid-filled blister under the skin.<br>• A cutaneous ulcer from where the worm emerges.<br>• Reddened and swollen abscesses.	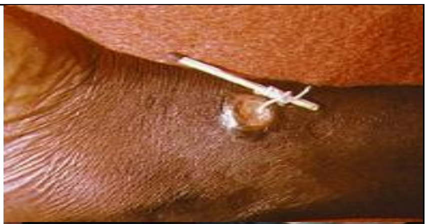<br>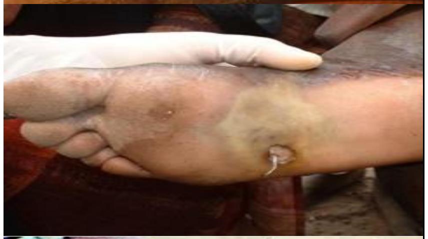<br>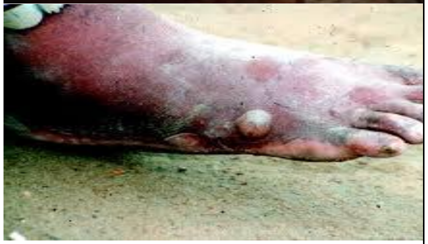
Lymphatic filariasis	Limbs, breast, genitals	Elephantiasis of the body's extremities including the legs, arms, breast and the male genitalia.	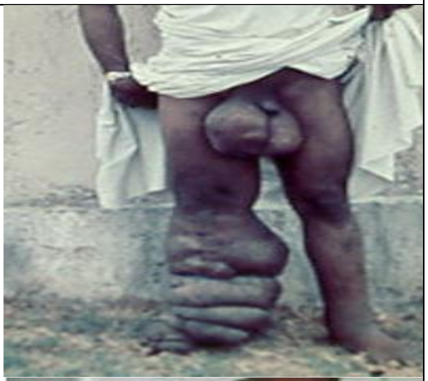<br>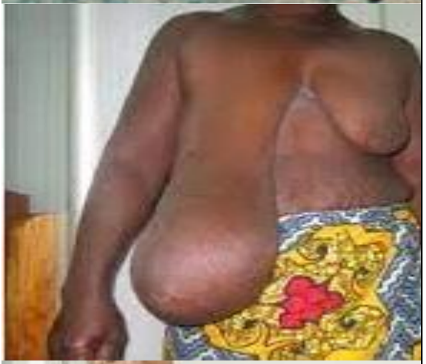<br>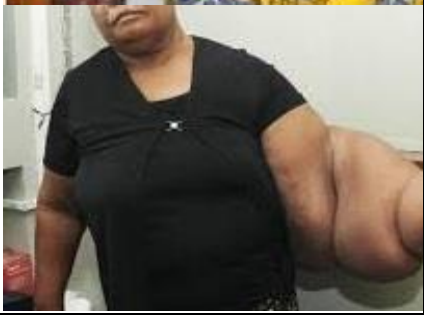

Onchocerc hiasis	Skin, eyes	• Acute papular onchode rmatitis- Widespread eczema-like rash with multiple small itchy papules affecting the face, trunk, and extremities.<br>• Chronic papularonchodermatitis - Rash with scattered flat-topped papules and areas of hyperpigmentation typically affecting the shoulders, buttocks, and extremities.<br>• Lichenified onchoderm atitis- thickened scaly and hyperpigmented itc hy plaques typically affecting the lower extremities often with enlarged lymph nodes.<br>• Onchocercal atrophy- large areas of wrinkled thin, dry inelastic skin. Commonly affects buttocks and lower back (Lizard skin).<br>• Onchocercal depigment ation- also called “leopard skin”. Areas of pigment loss (leukoderma), with islands of normally pigmented skin surrounding hair follicl es. Often affects the shins in a symmetrical pattern.<br>• Palpable onchocercal nodules (oncocercoma)- Subcutaneous lumps found over bony prominences (contain the adult worms). The	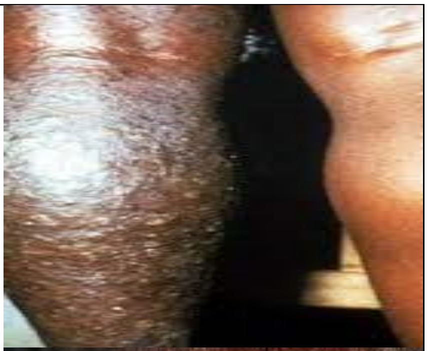 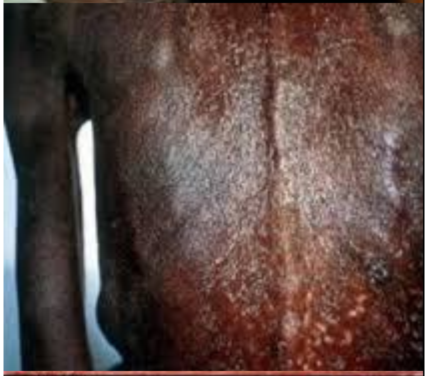 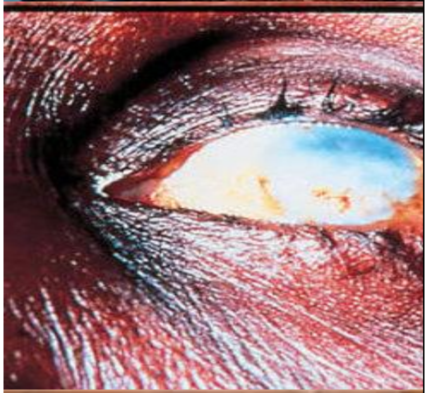 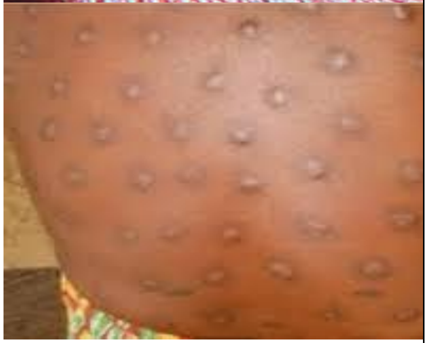

		subcutaneous nodules range in size from a few millimetres to several centimetres, and each contains 2 to 4 adult worms that can reach a length of 80 cm.<br>• Dry, scaly skin resembling ichthyosis(Lizard skin).<br>• Hanging groin– folds of inelastic atrophic skin in the groin associated with enlarged lymph nodes.<br>• Chronic keratitis and sclerosis affecting the clarity of the cornea. Glaucoma and wrinkle in the skin areas surrounding the eye.	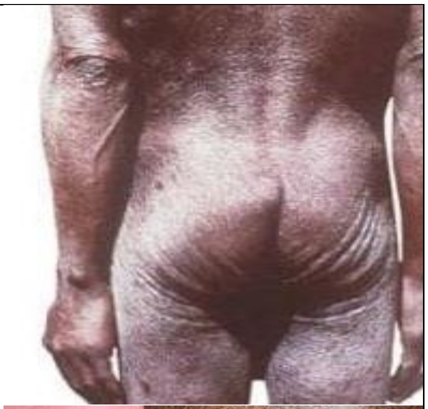 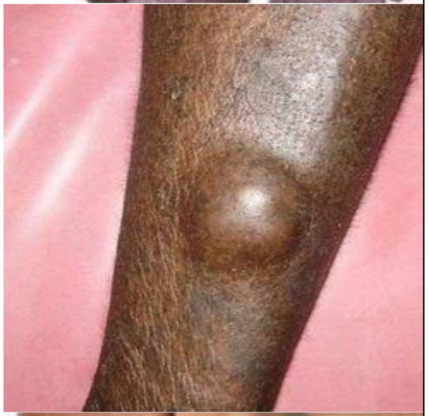 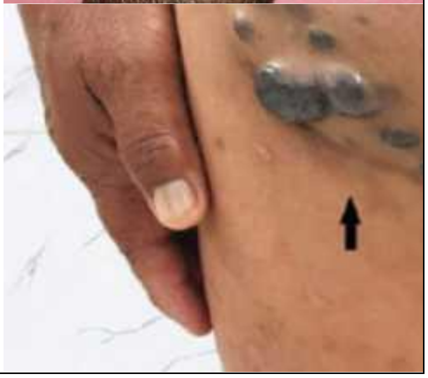

Ascariasis	Intestines	• The females have an average length of 30 cm and 3-6 mm in diameter.<br>• Males are smaller, ranging from 15-30 cm in length and 2-4 mm in diameter.<br>• Both sexes have an elongated, cylindrical body which tapers at both ends.<br>• In males the tail curves ventrally.<br>• Both sexes are cream-colored, sometimes with a pink tinge.	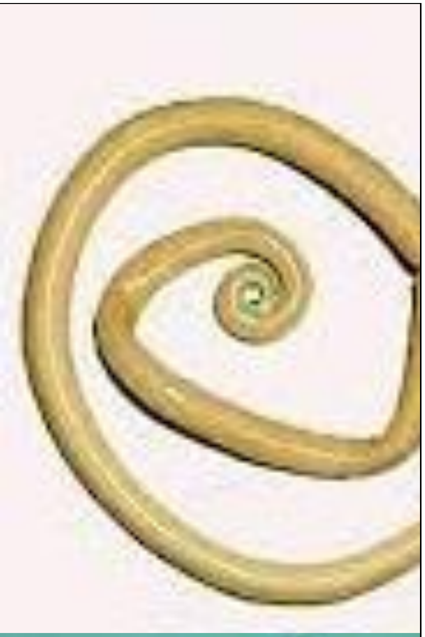<br>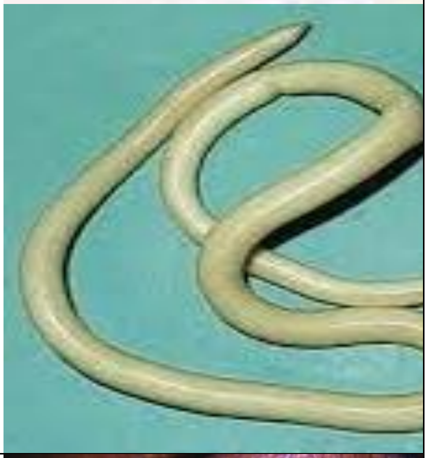
Trachoma	Eyes	• Trachomatous inflammation-Follicular: presence of at least five follicles in the upper tarsal conjunctiva.<br>• Trachomatous inflammation- Intense: pronounced inflammatory thickening of the upper tarsal conjunctiva, which obscures more than one-half of the normal tarsal vessels.<br>• Trachomatous scarring: the presence of easily visible white lines,	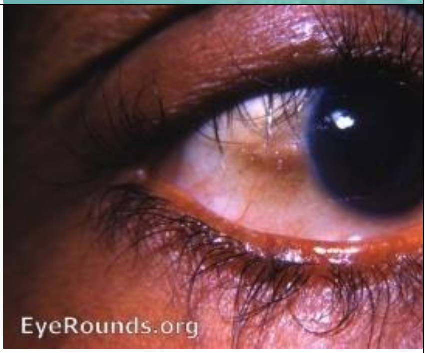

		bands, or sheets in the tarsal conjunctiva. The scarring may obscure the tarsal vessles.<br>• Trachomatous trichiasis: at least one eyelash that rubs the globe or evidence of recently removed in-turned eyelashes (epilation).<br>• Corneal opacity: the presence of an easily visible corneal opacity that obscures at least part of the pupillary margin.	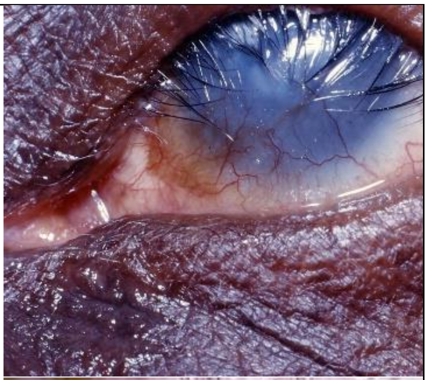 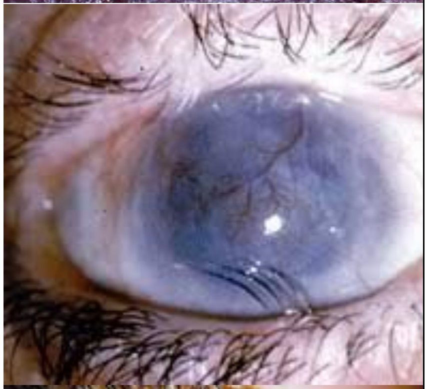 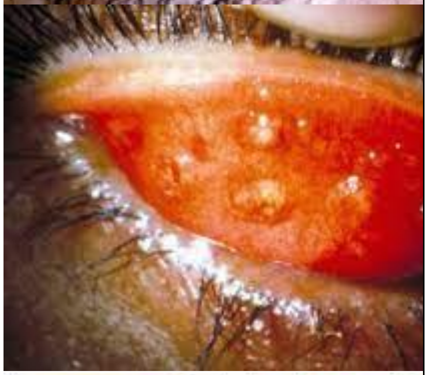 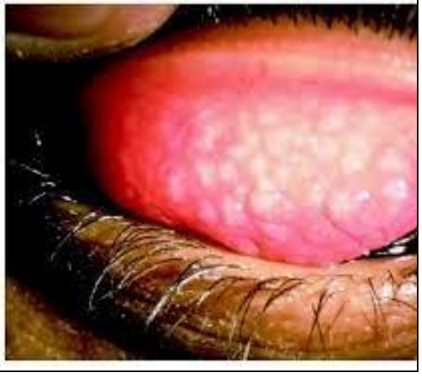

CIMS-NTDs RESEARCH WORKFLOW

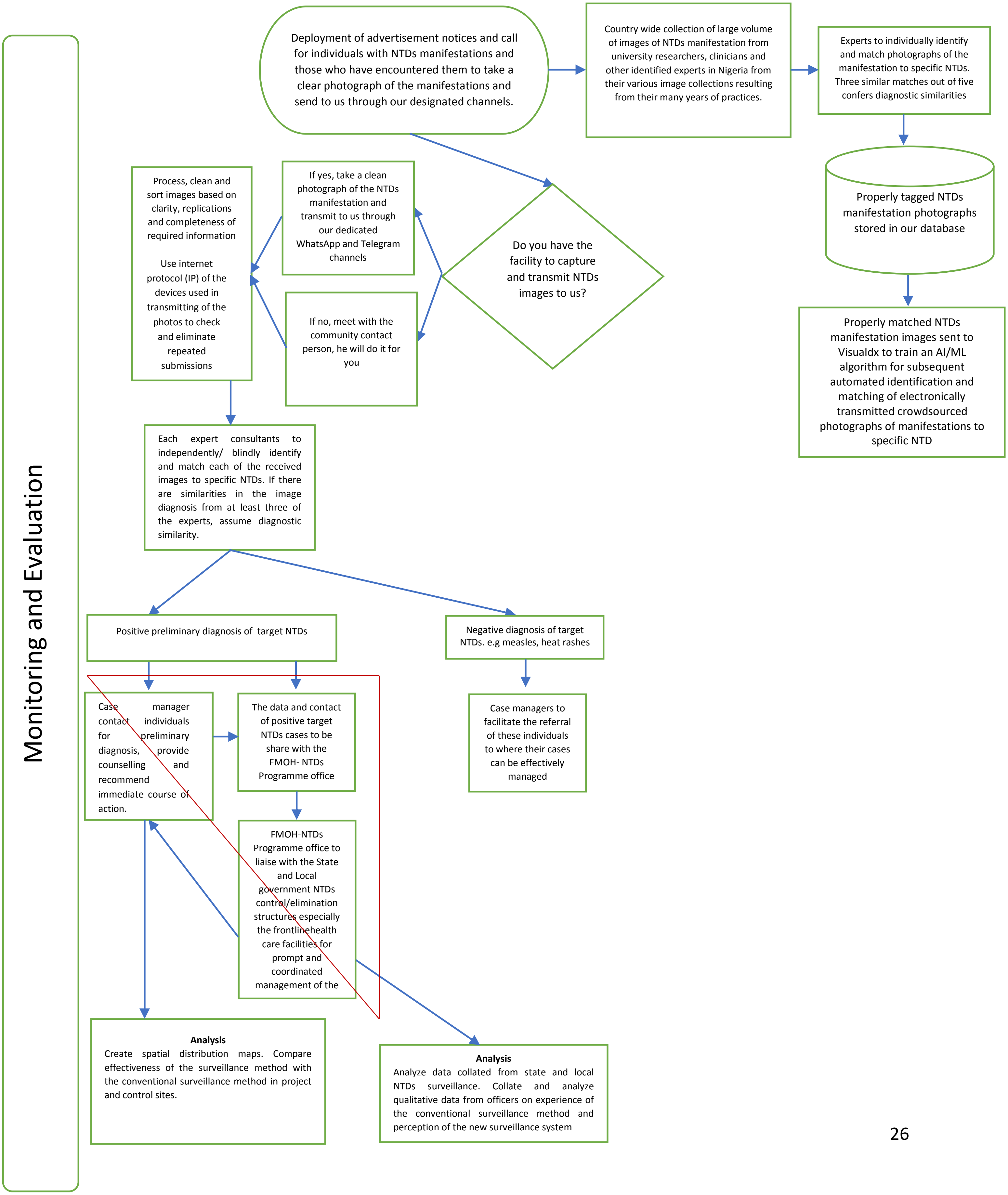

CIMS-NTDs RESEARCH WORKFLOW WITH ETHICAL CONSIDERATIONS (In Red)

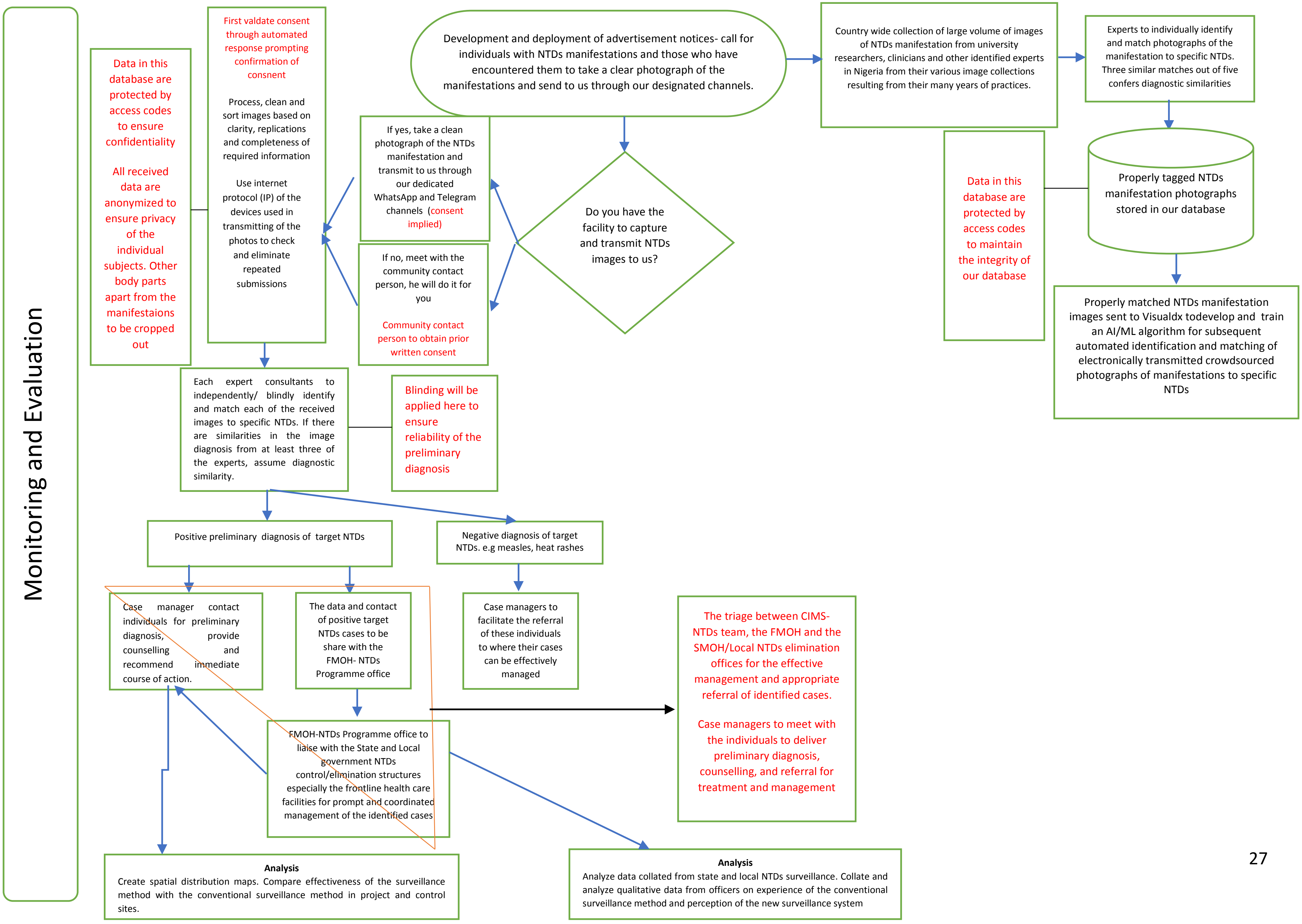

Supplement: S1 File — (ZIP) [file pone.0303179.s001.zip › PROTOCOL APPROVED BY NHREC _CIMS-NTDs_INV-048721-r.pdf]
