# Supplementary material for: Protocol for the crowdsourced image-based morbidity hotspot surveillance for neglected tropical diseases (CIMS-NTDs)
Source: PLoS One. 2024 May 10;19(5):e0303179. doi: 10.1371/journal.pone.0303179 (PMC11086871; doi:10.1371/journal.pone.0303179)
Supplement: S1 File — (ZIP) [file BMGF GRANT AWARD_CIMS-NTDs.pdf › BMGF GRANT AWARD_CIMS-NTDs.pdf]

BILL & MELINDA GATES foundation

PO Box 23350
Seattle, WA 98102, USA
V 206.709.3100
F 206.709.3180
www.gatesfoundation.org

November 11th, 2022

Uchechukwu M. Chukwuocha, PhD
Group Lead, Innovations and Technologies for Disease Control (INTEDeC) Research Group
Department of Public Health, Federal University of Technology, Owerri, Nigeria

Re: Grant No. INV-048721

Dear Dr. Chukwuocha,

Congratulations on the approval and activation of your grant! We look forward to working with you and your team over the coming months in our common pursuit of the grant project outcomes INV# 048721 “Crowdsourced Image-based Morbidity Hotspot Surveillance for NTDs".

We want to take this opportunity to share a few notes about contact information and roles, and communication guidelines. We are happy to review these details together by phone, and if you’re amenable, we’d like to schedule a kickoff call (details below).

Key Foundation Contacts: The Primary Program Officer, Lungi Okoko, (lungi.okoko@gatesfoundation.org) is your main point of contact and will work with you throughout the life of the grant to monitor progress and to provide thought partnership where requested. Let Mr. Okoko know right away if any issues or changes arise that may impact your ability to achieve the stated outcomes of the work, including changes to resources or your organization’s leadership.

The Program Coordinator, Elizabeth Hankins, (elizabeth.hankins@gatesfoundation.org) coordinates reviews and approvals of grant proposals, any grant amendments, reports, and payments; answers questions related to these and more administrative aspects of your grant. Please copy Mrs. Hankins in emails if requesting changes to your grant.

While you may on occasion hear from other members of our team on specific matters, when in doubt, you can always connect with us to route your questions appropriately. Of course, we encourage you to communicate openly with us about the progress of your grant, our partnership with you, or any questions/concerns that may arise during the course of your grant

Kickoff Call: We will be in touch shortly with some options for a kickoff call. Please aim for an hour. Let us know which options works best for your team and who else (if any) should be included in the Teams Meeting invitation. The proposed kick-off call agenda is as follows:
· Introductions (All)
· Investment details, key contacts, and how we work together
· Grant agreement and agreement deliverables overview
· Progress Reports, Final Reports, Deliverables & Outcomes
· Q&A; Outstanding items.

Finally, on behalf of the Bill & Melinda Gates Foundation, we are delighted to be working with you and look forward to a mutually beneficial partnership.

Sincerely,

Lungi Okoko
Senior Program Officer, Data Use
Neglected Tropical Diseases, Global Health
