# Supplementary material for: Protocol for the crowdsourced image-based morbidity hotspot surveillance for neglected tropical diseases (CIMS-NTDs)
Source: PLoS One. 2024 May 10;19(5):e0303179. doi: 10.1371/journal.pone.0303179 (PMC11086871; doi:10.1371/journal.pone.0303179)
Supplement: Attachment — Submitted filename: HOM_PONE-D-23-31987_reviewer.pdf [file pone.0303179.s002.pdf]

PLOS ONE

Crowdsourced Image-Based Morbidity Hotspot Surveillance for Neglected Tropical Diseases (CIMS-NTDs): Study Protocol

--Manuscript Draft--

**Manuscript Number:**	PONE-D-23-31987
**Article Type:**	Study Protocol
**Full Title:**	Crowdsourced Image-Based Morbidity Hotspot Surveillance for Neglected Tropical Diseases (CIMS-NTDs): Study Protocol
**Short Title:**	Crowdsourced Image-Based Morbidity Hotspot Surveillance for NTDs
**Corresponding Author:**	Uchechukwu Madukaku Chukwuocha<br>Federal University of Technology Owerri School of Health Technology<br>Owerri, Imo NIGERIA
**Keywords:**	Neglected Tropical Diseases; new surveillance method; crowdsourcing; image-based
**Abstract:**	Introduction<br>Effective surveillance and targeted deployment of interventions are essential components of strategies towards the elimination of Neglected Tropical Diseases (NTDs). Traditional surveillance systems are, however, time-consuming, expensive, and do not cover the entire population. One of the primary reasons is the inability of surveillance officers to visit communities due to movement restrictions. Therefore, it is crucial to develop and test an improved surveillance method that leverages smartphone technology and mobile communication networks. This method is envisaged to simultaneously conduct surveillance for multiple NTDs, making it cost-effective, efficient, and reliable. Additionally, it will actively involve NTDs subjects and their communities, making it effective even in situations with restricted movement. Furthermore, it will be invaluable for post-elimination surveillance.<br>Methods and Analysis<br>The surveillance method will involve sensitizing NTDs subjects and members of their communities to take photographs of NTDs manifestations upon notice using smartphones and to transmit the images along with other essential demographic data to a backend server through dedicated WhatsApp or Telegram accounts. The received images will be cleaned and sorted by the backend staff, who will then transmit them to a group of experts for image identification and matching to specific NTDs. The manifestation photographs which will be received by the consent of the sender will only show the body part with the manifestation without the faces or any other identification features. This is to ensure the privacy of the subjects. The study will last for eighteen months and will include activities like advocacy, community engagement, and trainings. Identified and matched photo-data together with geocoordinates information will be used to map the NTDs morbidity hotspots using ArcGIS while the accompanying metadata will be used to generate geographic and demographic distributions of various NTDs in the study sites. Additionally, NTDs prevalence data from the control states will be obtained during the period of the pilot study for the purpose of comparison to determine the effectiveness and efficiency of the CIMS-NTDs method against the traditional NTDs surveillance methods.<br>Ethics and Dissemination<br>The project was issued ethical approval (NHREC Approval Number NHREC/01/01/2007-16/01/2023) and the protocol was approved (NHREC Protocol Number NHREC/01/01/2007- 28/11/2022) by the National Health Research Ethics Committee of Nigeria (NHREC), Federal Ministry of Health, Abuja Nigeria. Findings will be reported to the study communities, the funding body, the World Health Organization, Nigerian Federal and States Ministries of Health to inform policy. Findings will be published in peer-reviewed journals and presented in both local and international scientific conferences.
**Order of Authors:**	Uchechukwu Madukaku Chukwuocha
	Christopher Sule Oyamienlen
	Ayoola Oluwaseun Bosede

Powered by Editorial Manager® and ProduXion Manager® from Aries Systems Corporation

	Ikechukwu Nosike Dozie
**Additional Information:**	
**Question**	**Response**
**Financial Disclosure**<br><br>Enter a financial disclosure statement that describes the sources of funding for the work included in this submission. Review the submission guidelines for detailed requirements. View published research articles from *PLOS ONE* for specific examples.<br><br>This statement is required for submission and **will appear in the published article** if the submission is accepted. Please make sure it is accurate.<br><br>**Funded studies**<br>Enter a statement with the following details:<br>• Initials of the authors who received each award<br>• Grant numbers awarded to each author<br>• The full name of each funder<br>• URL of each funder website<br>• Did the sponsors or funders play any role in the study design, data collection and analysis, decision to publish, or preparation of the manuscript?<br><br>Did you receive funding for this work?	Yes
Please add funding details.<br>as follow-up to "**Financial Disclosure**<br><br>Enter a financial disclosure statement that describes the sources of funding for the work included in this submission. Review the submission guidelines for detailed requirements. View published research articles from *PLOS ONE* for specific examples.<br><br>This statement is required for submission and **will appear in the published article** if the submission is accepted. Please make sure it is accurate.	UMC received the grant. The grant number is INV-048721. The funder is Bill and Melinda Gates Foundation https://www.gatesfoundation.org/<br>The funder did not play any role in the design, analysis, decision to publish or preparation of the manuscript.

**Funded studies**<br>Enter a statement with the following details:<br>• Initials of the authors who received each award<br>• Grant numbers awarded to each author<br>• The full name of each funder<br>• URL of each funder website<br>• Did the sponsors or funders play any role in the study design, data collection and analysis, decision to publish, or preparation of the manuscript?<br><br>Did you receive funding for this work?"	
Please select the country of your main research funder (please select carefully as in some cases this is used in fee calculation).<br>as follow-up to "**Financial Disclosure**<br><br>Enter a financial disclosure statement that describes the sources of funding for the work included in this submission. Review the submission guidelines for detailed requirements. View published research articles from *PLOS ONE* for specific examples.<br><br>This statement is required for submission and **will appear in the published article** if the submission is accepted. Please make sure it is accurate.<br><br>**Funded studies**<br>Enter a statement with the following details:<br>• Initials of the authors who received each award<br>• Grant numbers awarded to each author<br>• The full name of each funder<br>• URL of each funder website<br>• Did the sponsors or funders play any role in the study design, data collection and analysis, decision to publish, or preparation of the manuscript?<br><br>Did you receive funding for this work?"	UNITED STATES - US
**Competing Interests**	The authors have declared that no competing interests exist

Powered by Editorial Manager® and ProduXion Manager® from Aries Systems Corporation

Use the instructions below to enter a competing interest statement for this submission. On behalf of all authors, disclose any competing interests that could be perceived to bias this work—acknowledging all financial support and any other relevant financial or non-financial competing interests.<br><br>This statement is required for submission and **will appear in the published article** if the submission is accepted. Please make sure it is accurate and that any funding sources listed in your Funding Information later in the submission form are also declared in your Financial Disclosure statement.<br><br>View published research articles from *PLOS ONE* for specific examples.<br><br>**NO authors have competing interests**<br><br>Enter: *The authors have declared that no competing interests exist.*<br><br>**Authors with competing interests**<br><br>Enter competing interest details beginning with this statement:<br><br>*I have read the journal's policy and the authors of this manuscript have the following competing interests: [insert competing interests here]*<br><br>* typeset	
**Ethics Statement**<br><br>Enter an ethics statement for this submission. This statement is required if the study involved:<br><br>• Human participants<br>• Human specimens or tissue<br>• Vertebrate animals or cephalopods<br>• Vertebrate embryos or tissues	Ethical (NHREC Approval Number NHREC/01/01/2007-16/01/2023) and Protocol (NHREC Protocol Number NHREC/01/01/2007- 28/11/2022) approvals were given by the National Health Research Ethics Committee of Nigeria (NHREC), Federal Ministry of Health, Abuja Nigeria. Also, approval was obtained from the Institutional Review Board of the Federal University of Technology, Owerri, Nigeria. Our advertisements will bear a brief description of the project, its intentions and the role of potential participants in lay language, and request that only those who give consent to participate should transmit images of NTDs manifestations (without their faces) and accompanying demographics (without their names). To further validate informed consent, there will be an automated response from our WhatsApp and Telegram platforms prompting those

Powered by Editorial Manager® and ProduXion Manager® from Aries Systems Corporation

• Field research<br><br>Write "N/A" if the submission does not require an ethics statement.<br><br>General guidance is provided below. Consult the submission guidelines for detailed instructions. **Make sure that all information entered here is included in the Methods section of the manuscript.**	who transmit images/ data to confirm their consent and permission to use their data in this research. Additionally, our community contact persons who will assist those without smart devices to take photographs of the images and transmit will collect informed written consent from such subjects. For the children and vulnerable groups, their parents and care givers will be allowed to provide the consent on their behalf with the children assenting to it. Hard copies of signed consent forms will be collected from our Community Contact Persons and stored in a secure steel cabinet while electronic consent will be stored in our password secured electronic data base.

**Format for specific study types**<br><br>**Human Subject Research (involving human participants and/or tissue)**<br>• Give the name of the institutional review board or ethics committee that approved the study<br>• Include the approval number and/or a statement indicating approval of this research<br>• Indicate the form of consent obtained (written/oral) or the reason that consent was not obtained (e.g. the data were analyzed anonymously)<br><br>**Animal Research (involving vertebrate animals, embryos or tissues)**<br>• Provide the name of the Institutional Animal Care and Use Committee (IACUC) or other relevant ethics board that reviewed the study protocol, and indicate whether they approved this research or granted a formal waiver of ethical approval<br>• Include an approval number if one was obtained<br>• If the study involved *non-human primates*, add *additional details* about animal welfare and steps taken to ameliorate suffering<br>• If anesthesia, euthanasia, or any kind of animal sacrifice is part of the study, include briefly which substances and/or methods were applied<br><br>**Field Research**<br><br>Include the following details if this study involves the collection of plant, animal, or other materials from a natural setting:<br>• Field permit number<br>• Name of the institution or relevant body that granted permission	
**Data Availability**<br><br>Authors are required to make the data underlying their research findings fully available, without restriction.<br><br>If the manuscript reports pilot or preliminary results, the data underlying those results must be made available at the time of publication. A Data Availability	N/A - No results are reported

Powered by Editorial Manager® and ProduXion Manager® from Aries Systems Corporation

Statement describing where the data can be found is required at submission. Your answers to this question constitute the Data Availability Statement and will be published in the article, if accepted. When legal or ethical requirements prohibit public data sharing, authors must indicate how researchers may obtain access to the data. See the PLOS Data Policy and FAQ for detailed information.<br><br>**Important:** Stating 'data available on request from the author' is not sufficient. If your data are only available upon request, select 'No' for the first question and explain your situation in the text box.<br><br>For protocols without pilot or preliminary data, authors are strongly encouraged to state how they plan to share research data from their study when it is completed or published.<br><br>If your study includes pilot data, will they be made fully available without restriction?	
**Describe where the data may be found in full sentences. If you are copying our sample text, replace any instances of XXX with the appropriate details.**<br><br>• If **no pilot data are reported** in the manuscript, please enter the following: *No datasets were generated or analysed during the current study. All relevant data from this study will be made available upon study completion.*<br>• If the manuscript reports pilot data **held in a public repository**, include URLs, accession numbers or DOIs. If this information will only be available after acceptance, indicate this by ticking the box below. For example: *All XXX files are available from the XXX database (accession number(s) XXX, XXX.).*<br>• If the manuscript reports pilot data contained **within the manuscript and/or**	No datasets were generated or analysed during the current study. All relevant data from this study will be made available upon study completion.

**Supporting Information files**, enter the following:<br>*All relevant data are within the manuscript and its Supporting Information files.*<br>• If neither of these applies but you are able to provide **details of access elsewhere**, with or without limitations, please do so. For example:<br><br>*Data cannot be shared publicly because of [XXX]. Data are available from the XXX Institutional Data Access / Ethics Committee (contact via XXX) for researchers who meet the criteria for access to confidential data.*<br><br>*The data underlying the results presented in the study are available from (include the name of the third party and contact information or URL).*<br>• This text is appropriate if the data are owned by a third party and authors do not have permission to share the data.<br><br>For study protocols with data management or sharing plans, authors are encouraged to briefly describe in their Data Availability Statement how data they generate will be made accessible when the study is completed. For example:<br>• Deidentified research data will be made publicly available when the study is completed and published.<br><br>* typeset	
Additional data availability information:	Tick here if your circumstances are not covered by the questions above and you need the journal's help to make your data available.

Manuscript
Click here to access/download;Manuscript;CIMS-NTDs protocol_PlosOne.docx

Crowdsourced Image-Based Morbidity Hotspot Surveillance for Neglected Tropical Diseases (CIMS-NTDs): Study Protocol

Uchechukwu Madukaku Chukwuocha, Christopher Sule Oyamienlen, Ayoola Oluwaseun Bosede, Ikechukwu Nosike Simplicius Dozie

Innovations and Technologies for Disease Control Research Group, Department of Public Health, Federal University of Technology, Owerri, Nigeria.

Correspondence to uchukwuocha@gmail.com

Funding

This work is supported by a grant from the Bill and Melinda Gates Foundation (Grant no.: INV-048721)

Competing interests

None declared

Data availability

The study data will be made available to the funding organization upon the completion of the study, it will also be available to journals and partners for the purpose of research findings publication.

Abstract

Introduction

Effective surveillance and targeted deployment of interventions are essential components of strategies towards the elimination of Neglected Tropical Diseases (NTDs). Traditional surveillance systems are, however, time-consuming, expensive, and do not cover the entire population. One of the primary reasons is the inability of surveillance officers to visit communities due to movement restrictions. Therefore, it is crucial to develop and test an improved surveillance method that leverages smartphone technology and mobile communication networks. This method is envisaged to simultaneously conduct surveillance for multiple NTDs, making it cost-effective, efficient, and reliable. Additionally, it will actively involve NTDs subjects and their communities, making it effective even in situations with restricted movement. Furthermore, it will be invaluable for post-elimination surveillance.

Methods and Analysis

The surveillance method will involve sensitizing NTDs subjects and members of their communities to take photographs of NTDs manifestations upon notice using smartphones and to transmit the images along with other essential demographic data to a backend server through dedicated WhatsApp or Telegram accounts. The received images will be cleaned and sorted by the backend staff, who will then transmit them to a group of experts for image identification and matching to specific NTDs. The manifestation photographs which will be received by the consent of the sender will only show the body part with the manifestation without the faces or any other identification features. This is to ensure the privacy of the subjects. The study will last for eighteen months and will include activities like advocacy, community engagement, and trainings. Identified and matched photo-data together with geocoordinates information will be used to map the NTDs morbidity hotspots using ArcGIS while the accompanying metadata will be used to generate geographic and demographic distributions of various NTDs in the study sites. Additionally, NTDs prevalence data from the control states will be obtained during the period of the pilot study for the purpose of comparison to determine the effectiveness and efficiency of the CIMS-NTDs method against the traditional NTDs surveillance methods.

Ethics and Dissemination

The project was issued ethical approval (NHREC Approval Number NHREC/01/01/2007-16/01/2023) and the protocol was approved (NHREC Protocol Number NHREC/01/01/2007-28/11/2022) by the National Health Research Ethics Committee of Nigeria (NHREC), Federal Ministry of Health, Abuja Nigeria. Findings will be reported to the study communities, the funding body, the World Health Organization, Nigerian Federal and States Ministries of Health to inform policy. Findings will be published in peer-reviewed journals and presented in both local and international scientific conferences.

INTRODUCTION

Neglected Tropical Diseases (NTDs) have been designated as targets for elimination by the World Health Organization (WHO) in the NTDs Road Map 2021-2030 [1]. In 2022, the Kigali Declaration on NTDs was issued to galvanize political support and secure commitments aimed at achieving Sustainable Development Goal 3 (SDG3: Good Health and Well-being) targets related to NTDs and aligning with the objectives outlined in the WHO's Neglected Tropical Disease Roadmap [2]. NTDs constitute a group of debilitating and often overlooked diseases that primarily affect the world's most impoverished populations [1,3,4]. These diseases encompass a diverse range of conditions, including but not limited to lymphatic filariasis, onchocerciasis, schistosomiasis, soil-transmitted helminthiasis, and trachoma, to name a few. They share a common characteristic in their tendency to thrive in marginalized communities with limited access to basic healthcare, clean water, and sanitation facilities. The global burden of NTDs is staggering, with an estimated 1.7 billion people affected, many of whom live in low- and middle-income countries [1]. These diseases not only cause immense suffering but also perpetuate the cycle of poverty, hindering economic development and preventing affected individuals from reaching their full potential. The consequences extend beyond health, impacting education, workforce productivity, and overall societal well-being [5].

The WHO's bold NTDs Road Map 2021-2030 outlines a strategic framework for the control, elimination, and eradication of these diseases, with the ultimate goal of achieving a world free from NTDs-related suffering by the end of the decade[3]. While significant progress has been made in recent years, considerable challenges persist. Traditional surveillance methods, often reliant on healthcare workers physically visiting communities, are resource-intensive and geographically limited. This limitation has been particularly evident during crises such as the

COVID-19 pandemic, civil unrest, and natural disasters, when access to affected regions becomes severely restricted. Effective surveillance and precise intervention strategies are indispensable components in the pursuit of NTDs elimination.

Addressing these challenges requires innovative solutions that can augment existing surveillance efforts, reach underserved populations, and provide timely data for decision-making. Notably, recent experiences have demonstrated that crowdsourced data-based surveillance methods offer a cost-effective, real-time, scalable, and efficient means to enhance malaria surveillance [6]. It is within this context that this proposed project seeks to introduce a transformative approach to NTDs surveillance—one that leverages the power of crowdsourcing, smartphone technology, and artificial intelligence to create a dynamic and responsive system capable of delivering real-time insights into the distribution and prevalence of NTDs. By this, it is aimed that the global effort to combat NTDs will be bolstered, offering a cost-effective and scalable alternative that can be integrated into ongoing programs and bring the world closer to the realization of a world free from the burden of NTDs. Building on these insights, the proposed project aims to make a substantial contribution to achieving the WHO's NTDs elimination targets. The development of a crowdsourced image-based morbidity surveillance method to enhance the monitoring of NTDs is proposed. This method will be realized through the following objectives;

First, the development and validation of an electronically transmitted crowdsourced photograph method for enhanced NTDs surveillance. This objective involves educating affected individuals and community members on capturing images of NTDs manifestations upon notice using their smart devices. These images, along with relevant demographic data, will be transmitted to a designated phone number via social media handles. Subsequently, the collected data will be compiled in an electronic database, and experts will identify and categorize the images according

to specific NTDs. Location data will be used to map the distribution of these NTDs, aiding policymakers in better planning and targeted interventions. This approach can also facilitate post-elimination surveillance, as community members will be empowered to report potential cases swiftly.

Secondly, the development and validation of a feedback mechanism and personalized care for NTD subjects: Leveraging the database of telephone numbers collected through image transmission, this objective enables the delivery of personalized feedback on preliminary diagnoses and recommendations for immediate morbidity management and disability prevention. Non-clinical support, such as guidance on hygiene practices and transmission avoidance behaviors, can also be provided. Additionally, subjects can receive information on when, where, and how to access available interventions.

Finally, the development and training of an Artificial Intelligence (AI)/Machine Learning (ML) algorithm. An AI-driven matcher and mobile application will be created. This tool, based on the database of categorized images and NTDs knowledge, will assist healthcare personnel in differential diagnosis and real-time clinical management or referral advice at health facilities. While field testing of the AI mobile application falls outside the scope of this project, it lays the groundwork for future deployment.

It is anticipated that this project phase will yield a timely, cost-effective, population-inclusive, feasible, and efficient alternative to traditional NTDs surveillance methods. It holds promise for scalability, integration into ongoing NTDs elimination efforts and health systems, enhanced large-scale surveillance of NTDs in the country and the sub-Saharan African region, simultaneous monitoring of multiple NTDs, improved diagnosis, case management, prevention, and, ultimately,

significant contributions toward NTDs elimination. Furthermore, this method will prove invaluable for post-elimination surveillance, enabling the prompt identification of residual cases or underserved areas and groups to prevent resurgence. Future endeavors will explore full automation and digitalization of the entire image-based surveillance and response process with minimal human intervention, utilizing artificial intelligence, machine learning, cloud computing tools, and further personalization for affected subjects.

MATERIALS AND METHODS

Aim of the study

Traditional NTD surveillance systems are often plagued by inefficiencies, as they are time-consuming, costly, and frequently fail to encompass the entire population. Moreover, these methods prove ineffective in circumstances where access to NTD-endemic areas is restricted, such as during the COVID-19 pandemic, civil unrest, or natural disasters. Currently, Nigeria lacks an active surveillance system for NTDs, relying instead on passive reporting. This passive approach involves generating NTD prevalence data when cases happen to present themselves at healthcare facilities for complaints or treatment. Following diagnosis confirmation, the cases are reported to Local Government Area Disease Surveillance and Notification Officers (DSNO), who subsequently forward the reports to State-level DSNOs through the Integrated Disease Surveillance and Response System (IDSR) [7]. However, this passive surveillance method falls short in supporting the mapping of NTDs prevalence required for targeted interventions, aligning with the global goal of eliminating these diseases by 2030. Furthermore, many NTD cases in

Nigeria go unreported at healthcare facilities due to the stigma associated with these conditions and the potential shame they may bring upon those affected.

To address these challenges, the Innovations and Technologies for Diseases Control (INTEDeC) research group at the Federal University of Technology Owerri has developed a Crowdsourced Image-Based Morbidity Hotspot Surveillance for NTDs (CIMS-NTDs) in Nigeria. This innovative surveillance approach harnesses smartphone technology, empowering community members to capture clear images of NTDs manifestations when they arise and transmit them, along with complete demographic data, to a designated WhatsApp or Telegram number. These photographs are received and compiled into a database, where a team of experts with extensive experience in NTDs research and practice, including parasitologists and dermatologists, will match each image to specific NTDs as a preliminary diagnosis. The results derived from this photo-data analysis will be employed to generate spatial maps depicting NTDs prevalence across the country, facilitating targeted interventions. Importantly, this method allows individuals who are uncomfortable visiting healthcare facilities due to fear of stigmatization and shame, which often accompany NTDs, to report their cases while ensuring their privacy.

This study aims to evaluate the effectiveness, feasibility, and potential impact of the CIMS-NTDs surveillance method in improving NTDs surveillance and advancing the progress towards the global elimination of these diseases by 2030.

Study setting

The project will be carried out in Nigeria. Nigeria has a tropical climate with variable rainy and dry seasons, depending on location. It is hot and wet most of the year in the southeast but dry in the southwest and farther inland. Nigeria is one of the most densely populated nations in Africa,

the most populous country in the African continent, and the seventh-most populous nation in the world. Hence, Nigeria is a likely target market for mobile phone, internet, and telecommunication businesses, and as such has influenced many Nigerians to becoming smartphones owners. It is estimated that about 90% of the country's population are mobile phone owners [8]. Nigeria has a significant amount of cultural diversity, due to its more than 250 distinct ethnic groups, more than 500 languages, and the variety of traditions practiced by each of them. Nigeria's economic situation is such that more than 40% of the population lives in poverty, with poor social and healthcare infrastructure. This situation together with environmental and climatic factors encourage the spread of a variety of diseases, including neglected tropical diseases. Nigeria bears the second highest global burden of NTDs [9].

Sample Size, Inclusion and Exclusion Criteria

Six pilot states will be purposively selected based on available data from the Nigeria's Federal Ministry of Health, on the endemicity of the target NTDs. Three of these states will be randomly selected as the intervention states while the other three states will serve as controls. An additional criterion for selection will be, endemic states where the Partner NGOs are already operating. NTDs surveillance data will be collected from State NTDs programme offices of both the intervention and control states. These data will be used to compare outcomes between our method and the conventional method within the intervention states as well as with the control states. The study will be a population-based study, and every available member of the project implementation sites shall be recruited into the study.

Description of Processes

Figure 1 is the flowchart of the entire project process while figure 2 is a simple pictorial depiction of the CIMS-NTDs idea. The project process by timeline is presented below.

Inception meeting of project partners (Month 1)

This project will commence with a hybrid meeting (virtual and in-person) of all project partners. The project partners include the Federal Ministry of Health of Nigeria Programme Offices (NPO) for NTDs, VisualDX and NGOs- whose role will be majorly community mobilization, sensitization and follow up. This meeting will involve further familiarization of project partners, assessment of the project design, procedures and protocols, confirmation of assignment of roles as well as setting up the work flow/system for the project.

Designing the advertisement and selecting project sites (Month 1)

At the beginning of the project, we will initiate the process of designing advertisements and selecting project sites. This crucial phase involves the following key steps:

1. Photo and Literature Search: We will conduct an extensive search for photographs depicting the manifestations of the targeted Neglected Tropical Diseases (NTDs), including Onchocerciasis, Lymphatic filariasis, Trachoma, Buruli Ulcer, Guinea worm infection, Leprosy, and Ascariasis. These photos will serve as visual references for our awareness campaign.

2. Photo Validation: Selected photos will undergo validation by National Programme Office for NTDs officials, following the World Health Organization (WHO) standard operating procedures (SOP). This validation process ensures the accuracy and relevance of the chosen images.

3. Advertisement Content: The validated photos will be used to craft our study advertisement/notice. The content of the advertisement will include:

- Distinctive photos illustrating the manifestations of the targeted NTDs, accompanied by descriptions in both English and local languages relevant to the study locations.
- A call to action for individuals who either have NTDs manifestations or have encountered them to capture clear photographs of these manifestations using their smartphones. Consent from the subjects will be obtained, and the images will exclude the subjects' faces.
- Instructions to transmit the photographs, along with demographic information about the subject (including location, age, gender, occupation, marital status, and educational level), through designated social media channels such as WhatsApp and Telegram. Alternatively, community members lacking the necessary technology can utilize a designated community contact person (CCP), whose name and phone number will be provided on the advertisement.
- A notice indicating that the NPO will provide available intervention measures to those who comply with the request.

4. Multilingual Narratives: Advertisements will feature narratives in both English and the local languages spoken in the pilot locations to ensure broad accessibility and understanding.

Advertisement Materials: To disseminate this critical information effectively, we will produce the following advertisement materials:

- Signposts called ***C2SSS*** (Community Crowdsourced Surveillance Sensitization Signposts): These 8ft by 4ft signposts will be strategically placed in each study community.

- Posters called ***C2SSP*** (Community Crowdsourced Surveillance Sensitization Posters): Approximately 300 posters will be affixed in and around each pilot community to maximize visibility.
- Flyers called ***C2SSF*** (Community Crowdsourced Surveillance Sensitization Flyers): Around 1000 flyers will be distributed throughout the pilot communities, with an additional 300 flyers shared every three months to maintain ongoing awareness within the communities.

This phase sets the stage for community engagement, data collection, and the implementation of our crowd-sourced image-based morbidity surveillance method.

5. Selection of Pilot Study Sites

To ensure a robust and comprehensive study, we will follow a systematic process to select our pilot study sites;

a. Choice of Endemic States

We will begin by identifying states with a high prevalence of Neglected Tropical Diseases (NTDs) based on data provided by the Federal Ministry of Health (FMOH) NTDs Programme Office.

b. Study Site Categories

Project Implementation Sites: Among the identified states, three will be designated as project implementation sites, where we will actively implement and evaluate our crowdsourced image-based morbidity hotspot surveillance method.

Control Sites: The remaining three states will serve as control sites. In these states, we will obtain the usual NTDs surveillance data that will later be used as a basis for comparison with the data generated by the piloted surveillance method.

c. Selection of Local Government Areas (LGAs)

In the selected project implementation states, we will further narrow down our focus by selecting five Local Government Areas (LGAs) from each state. These LGAs will be chosen based on recommendations and NTDs prevalence data from the respective state's NTDs programme office.

d. Community/Ward Selection

From each of the five selected LGAs, we will choose three communities/wards to serve as our project pilot study sites.

This meticulous selection process allows us to gather data from both project implementation sites and control sites, ensuring a comprehensive assessment of the CIMS-NTDs' effectiveness. By comparing data from the two groups, we aim to provide valuable insights into the impact and potential benefits of our innovative approach.

Advocacy visits, engagement of local stakeholders and inception workshop (Month 2)

Stakeholders in the selected states, including officials from State and Local Government health authorities, religious and community leaders, as well as local and international NGOs supporting NTDs programmes in the intervention states, will be engaged to solicit their support in sensitizing and mobilizing community members, and in any other way that could help the project succeed.

Community Contact Persons (CCPs) will be selected from among suitable Community Directed Distributors (CDDs) of ivermectin or any suitable community member who can operate a

smartphone and will be domiciled in the community throughout the project's duration. These CCPs will serve as the liaison between the project team and the project community. They will assist in carrying out project sensitization, distributing project C2SSF, and posting C2SSP materials around the community after the team has departed. An inception workshop will be held to interact with and seek the opinions of key stakeholders regarding the project's purpose and procedures, which will be vital to secure community buy-in and for its successful execution.

Intervention communities' mobilization and sensitization (Month 3&4)

Following stakeholder engagement, mobilization and sensitization of communities in the intervention states will commence. This will be achieved by securing the support of the National NTDs Programme Office to collaborate with selected State NTDs Programme Officers. The project team will work with two state NTDs officers and two Local Government Area (LGA) NTDs officers, comprising the LGA's social mobilization officer and the LGA's NTDs focal person. The primary goal is to raise awareness about the project among community members, ensuring their active participation throughout its duration.

These initial engagement activities will be complemented by ongoing engagement throughout the project, promoting community ownership and allowing for adaptation to optimize project implementation. Additionally, there will be periodic advocacy visits to the project communities and continuous engagement with community stakeholders, both in-person and virtually, to ensure their involvement and commitment throughout the project, ultimately fostering community ownership.

During this community engagement and sensitization, advertisements/notices that have been designed will be produced after validation by the Federal Ministry of Health's national NTD

programme office and deployed in intervention states as follows; **C2SSS-** to be deployed at strategic locations such as community centers; **C2SSP-** to be posted at primary, secondary and tertiary health facilities, commuter parks, religious centers, markets and schools; **C2SSF-** to be distributed at all public gatherings within the communities including the market squares, healthcare facilities, churches and schools. Additional adverts/notices will be placed on local Radio stations. These are aimed at adequately sensitizing the public on the procedures and their involvement in the process.

Control states will continue with the traditional surveillance systems without the intervention.

Large volume collection of photographs of manifestations of NTDs and development of artificial intelligence (AI) driven matcher of images to specific NTDs (Month 4-7)

This activity will be conducted simultaneously with the project implementation in interventions communities (month 3&4). In the initial stages of the project, expert consultants with extensive experience in NTD research, along with officials from the National NTDs Programme Office under the Federal Ministry of Health and personnel from partner NGOs, will be requested to gather, validate, and categorize a substantial volume of photographs illustrating various stages of NTD manifestations prevalent in the project locations. Digital cameras will be employed for this purpose. Additionally, we will collaborate with researchers from universities, clinicians, and recognized NTD experts in Nigeria and other parts of the world to solicit their contributions of confirmed NTD cases' photographs from their diverse image collections. These collected photographs will form the dataset essential for creating and training the AI and Machine Learning (ML) algorithms. This algorithm will facilitate the automated identification and matching of electronically transmitted crowdsourced images with specific NTDs. Furthermore, this dataset,

combined with a comprehensive knowledge database, will serve as the foundation for developing a point-of-care differential diagnosis and management mobile application.

Before applying the AI/ML photo matching algorithm to crowdsourced images, we will rigorously test it on a set of test images and compare its results with the outcomes of expert matching processes to ensure its validity and diagnostic accuracy.

Reception, collation, processing and analysis of photo and demographic data, and other data (Month 4-15)

Transmitted photos and demographic data will be received and collated in a central electronic data base and protected with an access code. Photos will be processed and sorted based on clarity, replications and complete data. Internet Protocol (IP) of the devices used in the transmission of the photos will be checked to eliminate repeated submissions. All data will be anonymized so that they will not be linked to individuals. A data protection expert will be involved to ensure compliance with relevant data protection laws. Initially, we will work with our expert consultants to identify, confirm, and match crowdsourced images to specific NTDs, while work on development, training and validation of the AI/ ML algorithm is ongoing. As soon as the AI/ ML algorithm has been trained and validated to the extent of confidence required to screen and match transmitted images, it will then be applied to replace identification/ matching of images by expert consultants. Subsequently, therefore, the AI/ML will automatically identify and match crowdsourced photos to specific NTDs. Arc Map, a digital mapping software will thereafter be used to map the respective target NTDs using the identified/ matched photos together with the location data along with the GPS location imprints of the transmitted images. Demographic and geographic data will be analysed using statistical software packages like SPSS and R to determine geographic and demographic distribution of these NTDs. Towards the end of the photo or image

data collection, quantitative and qualitative techniques will be used to collect data from the subjects on their experience, acceptability, usability and feasibility of this crowdsourced image-based NTDs surveillance method. Data will also be collected from officers at local government NTDs programme offices on their experiences with the conventional NTDs surveillance method and possible perceptions about this new method.

Feedback on preliminary diagnosis and information on actions to take (Personalized/ Individual Care) (Month 4-15)

Concurrently with data reception, transmission, collation and processing, there will be feedback to subjects whose data were transmitted through the telephone numbers used in transmitting such data. This will be done as the respective photos are successfully matched to specific NTDs. The feedback will be on the preliminary diagnosis of their conditions, counselling, and provision of information on appropriate actions to take as well as follow up, which will be undertaken by the project case managers and officers who are physicians with experiences regarding NTDs. They will also visit the communities periodically to provide diagnosis physically and support to identified subjects. In conjunction with the FMOH's national NTDs programme and local authorities/ health facilities as well as the community contact persons, standard operating procedure (SOPs) for investigating, triaging between the Federal, State, Local Government NTD control/ elimination programme offices and responding to NTD cases that are confirmed remotely will be developed to ensure confirmatory diagnosis and appropriate treatment is provided. This will involve leveraging on existing NTDs control/ elimination structures which includes frontline health facilities in every Local Government where identified cases of NTDs will be managed and counselled, and referral to tertiary health facilities where more serious cases beyond the capacity of the frontline facilities will be handled. Community contact persons together with selected

community health workers at the local or community levels will additionally be trained as adherence counselors to track individuals who transmitted images and received feedback to ensure that they take recommended actions and assist in delivering specific messages against stigmatization.

Outcome Measures

At the conclusion of the pilot study, our primary focus will be on evaluating the effectiveness and efficiency of the CIMS-NTDs method in comparison to the conventional surveillance approach. To assess this, we will primarily examine the quantity and quality of NTDs data collected using the CIMS-NTDs method at the pilot study site. This data will then be compared with the NTDs prevalence information gathered through the traditional surveillance method, both within the pilot study locations and in the control locations. It is important to note that this comparison will exclusively consider NTDs data collected from both methods during the study period.

Data Management Plans

The majority of the data generated by the study will consist of photographs depicting various manifestations submitted by participants via WhatsApp or Telegram, along with accompanying metadata. Upon receipt, this data will be processed in our database, undergoing cleaning and organization before being transmitted to our experts for precise matching to specific NTDs. Stringent security measures will be in place to safeguard this data within the database. Upon the return of annotated results, they will be integrated into another section of the database, where they will be transformed into results and presented as preliminary diagnoses.

Types of Data and Planned Statistical Analyses

The data collected for this study will primarily encompass the following:

1. Photographs: These will be visual representations of NTD manifestations submitted by participants, providing valuable visual information for diagnosis.

2. Metadata: Accompanying information, such as timestamps, location data, and patient details, will help contextualize and organize the photographs.

3. Qualitative data from NTDs stakeholders in study locations

4. Data assessing the perception of NTDs subjects and the people in the study locations about their perception about CIMS-NTDs and the ease of reporting using the CIMS-NTDs method.

In this section, we outline our data analysis plan, which encompasses the utilization of received photo and accompanying metadata to develop incidence and prevalence data for NTDs in our study sites. This data will be disaggregated by age, sex, educational attainment, and occupation of the subjects, providing a comprehensive overview of the affected demographics. Additionally, we will gather information regarding the current management of NTDs among the subjects and present this information in a straightforward frequency distribution table.

To assess the efficacy of the CIMS-NTDs method compared to the traditional surveillance approach, we will collect NTDs prevalence data from control states and traditional NTDs surveillance prevalence data from intervention states. Our comparative analysis will consider various indicators, including but not limited to:

1. Proportion of suspected NTD cases (per disease) reported weekly/monthly.
2. Rate and timeliness of reporting of suspected cases.
3. Proportion of identified cases that received a response in the intervention states versus the control states.

4. Promptness of the response received.
5. Demographic and geographic distributions of identified cases per specific focus NTD.

For geographic analysis, we will employ Arc GIS to create spatial maps highlighting NTDs morbidity hotspots within the pilot study states. These maps will be generated based on the NTDs images provided by the affected individuals and their respective communities.

In addition to quantitative data, we will gather qualitative insights through key informant interviews with NTDs stakeholders in the pilot states. These interviews will focus on their experiences with the traditional NTDs surveillance method and their perceptions of the CIMS-NTDs method. Furthermore, we will assess community members' experiences and perceptions of the CIMS-NTDs method using structured, interviewer-administered questionnaires. Responses will be compiled, aggregated, and visually presented in charts for clarity. Experiences will be categorized as either "good," "poor," or "neutral," while perceptions will be reported as either "positive" or "negative."

This comprehensive data analysis plan will enable us to evaluate the effectiveness of the CIMS-NTDs method and its potential benefits over traditional surveillance approaches, ultimately contributing to the enhancement of NTDs control and management strategies.

Ethical Considerations and Informed Consent

Ethical (NHREC Approval Number NHREC/01/01/2007-16/01/2023) and Protocol (NHREC Protocol Number NHREC/01/01/2007- 28/11/2022) approvals were given by the National Health Research Ethics Committee of Nigeria (NHREC), Federal Ministry of Health, Abuja Nigeria. Also, approval was obtained from the Institutional Review Board of the Federal University of

Technology, Owerri, Nigeria. Our advertisements will bear a brief description of the project, its intentions and the role of potential participants in lay language, and request that only those who give consent to participate should transmit images of NTDs manifestations (without their faces) and accompanying demographics (without their names). To further validate informed consent, there will be an automated response from our WhatsApp and Telegram platforms prompting those who transmit images/ data to confirm their consent and permission to use their data in this research. Additionally, our community contact persons who will assist those without smart devices to take photographs of the images and transmit will collect informed written consent from such subjects. For the children and vulnerable groups, their parents and care givers will be allowed to provide the consent on their behalf with the children assenting to it. Hard copies of signed consent forms will be collected from our Community Contact Persons and stored in a secure steel cabinet while electronic consent will be stored in our password secured electronic data base.

DISCUSSION

This protocol presents a comprehensive description of an ongoing pilot study introducing an innovative surveillance method for Neglected Tropical Diseases (NTDs), aligning with the WHO's NTDs elimination agenda for 2030, as outlined in the NTDs Roadmap 2021-2030 (10). The surveillance method employed in this study breaks new ground by leveraging smartphone technology to empower community members. Participants are sensitized to capture clear images of NTDs manifestations and transmit them, along with their complete demographic data, via platforms such as WhatsApp or Telegram. This approach not only ensures the privacy of individuals reporting NTDs but also mitigates the potential stigmatization and shame often associated with visiting healthcare facilities for NTDs reporting [11]. Furthermore, it actively

engages subjects in the surveillance process, promoting inclusivity within the population. Simultaneously, it facilitates the surveillance of multiple NTDs, which is especially valuable for post-elimination monitoring [12].

A notable limitation of this project is the restricted access to smartphones and other smart devices among many residents in rural communities, which are often the areas most heavily affected by NTDs [13]. To address this limitation, a community contact person (CPP) is selected, trained, and provided with a smartphone if the individual does not have one. The CPP plays a pivotal role in taking photographs and transmitting them on behalf of individuals who wish to report NTDs cases but lack the necessary technology.

Dissemination of the knowledge and information acquired from the project will occur through diverse mechanisms. First, a dissemination seminar will be conducted to communicate project outcomes and information to stakeholders. Second, policy briefs will be developed and shared with decision-makers to facilitate timely implementation and decision-making [14]. Third, the findings will be made publicly available through scientific publications in open-access peer-reviewed journals and presentations at local and international scientific conferences. Fourth, project outcomes and information will be accessible on our organizational website, the Bill and Melinda Gates Foundation website, as well as the websites of other partner organizations. Additionally, key outcomes, outputs, and takeaways from the project will be shared on social media platforms such as Facebook, Twitter, and Instagram to enhance public awareness and engagement.

In conclusion, this pilot study's innovative approach to NTDs surveillance holds promise in significantly improving surveillance for NTDs and accelerating the WHO's attainable goals for NTDs elimination by 2030. Despite challenges related to smartphone accessibility in rural areas,

the inclusion of CPPs helps bridge this gap, ensuring equitable participation in the surveillance process.

AUTHORS CONTRIBUTIONS

UMC conceived the study, designed the protocol, provided the knowledge base for the study and is the principal investigator for the grant; CSO and AOB helped in the design and development of the protocol as well as the drafting of the initial manuscript; INSD revised the initial manuscript draft; all the authors read and approved the final draft of the manuscript.

ACKNOWLEDGMENTS

We would like to express our heartfelt gratitude to the following organizations and entities for their invaluable support and contributions to this research project;

The Bill and Melinda Gates Foundation for their generous funding, which made this study possible. The Nigeria Federal Ministry of Health NTD Programme Office for their collaboration and partnership throughout the project.

REFERENCES

1. WHO. Neglected Tropical Diseases (NTDs) [Internet]. Health Topics. 2022 [cited 2022 Nov 28]. p. 1. Available from: https://www.who.int/health-topics/neglected-tropical-diseases#tab=tab_1
2. Uniting to Combat Neglected Tropical Diseases Coalition. The Kigali Declaration, 2022 [Internet]. 2022 [cited 2023 May 27]. p. 1. Available from: https://unitingtocombatntds.org/kigali-declaration/
3. Malecela MN, Ducker C. A road map for neglected tropical diseases 2021–2030. Vol. 115, Transactions of the Royal Society of Tropical Medicine and Hygiene. Oxford University Press; 2021. p. 121–3.
4. Organization WH. Uniting to combat neglected tropical diseases. London Declar Neglected Trop Dis Uniting to Combat NTDs Haywards Heal UK. 2012;

5. Mitra AK, Mawson AR. Neglected tropical diseases: epidemiology and global burden. Trop Med Infect Dis. 2017;2(3):36.

6. Adekanmbi O, Oyewusi WF, Ogundepo E. Real-Time Crowdsourcing of Health Data in a Low-Income Country: A Case Study of Human Data Supply on Malaria First-Line Treatment Policy Tracking in Nigeria. In: CSW@ NeurIPS. 2020. p. 14–8.

7. Fall IS, Rajatonirina S, Yahaya AA, Zabulon Y, Nsubuga P, Nanyunja M, et al. Integrated Disease Surveillance and Response (IDSR) strategy: current status, challenges and perspectives for the future in Africa. BMJ Glob Heal. 2019;4(4):e001427.

8. Gilbert P. Nigerian internet and mobile penetration grows. Retrieved from Connect Africa website https//www Connect com/author asp. 2021;

9. Hotez PJ, Asojo OA, Adesina AM. Nigeria : '" Ground Zero "' for the High Prevalence Neglected Tropical Diseases. PLoS Negl Trop Dis. 2012;6(7).

10. WHO. Ending the neglect to attain Sustainable Development Goals: A road map for neglected tropical diseases 2021-2030. Geneva, Switzerland; 2021.

11. Wilsher E. The impact of Neglected Tropical Diseases, and their associated stigma, on people's basic capabilities. Durham University; 2011.

12. Fang Y, Zhang Y. Lessons from lymphatic filariasis elimination and the challenges of post-elimination surveillance in China. Infect Dis Poverty. 2019;8:1–10.

13. Gyapong JO, Gyapong M, Yellu N, Anakwah K, Amofah G, Bockarie M, et al. Integration of control of neglected tropical diseases into health-care systems: challenges and opportunities. Lancet. 2010;375(9709):160–5.

14. Pablos- Mendez A, Shademani R. Knowledge translation in global health. J Contin Educ Health Prof. 2006;26(1):81–6.

Figure

Click here to access/download;Figure;Figures_CIMS-NTDs protocol.docx

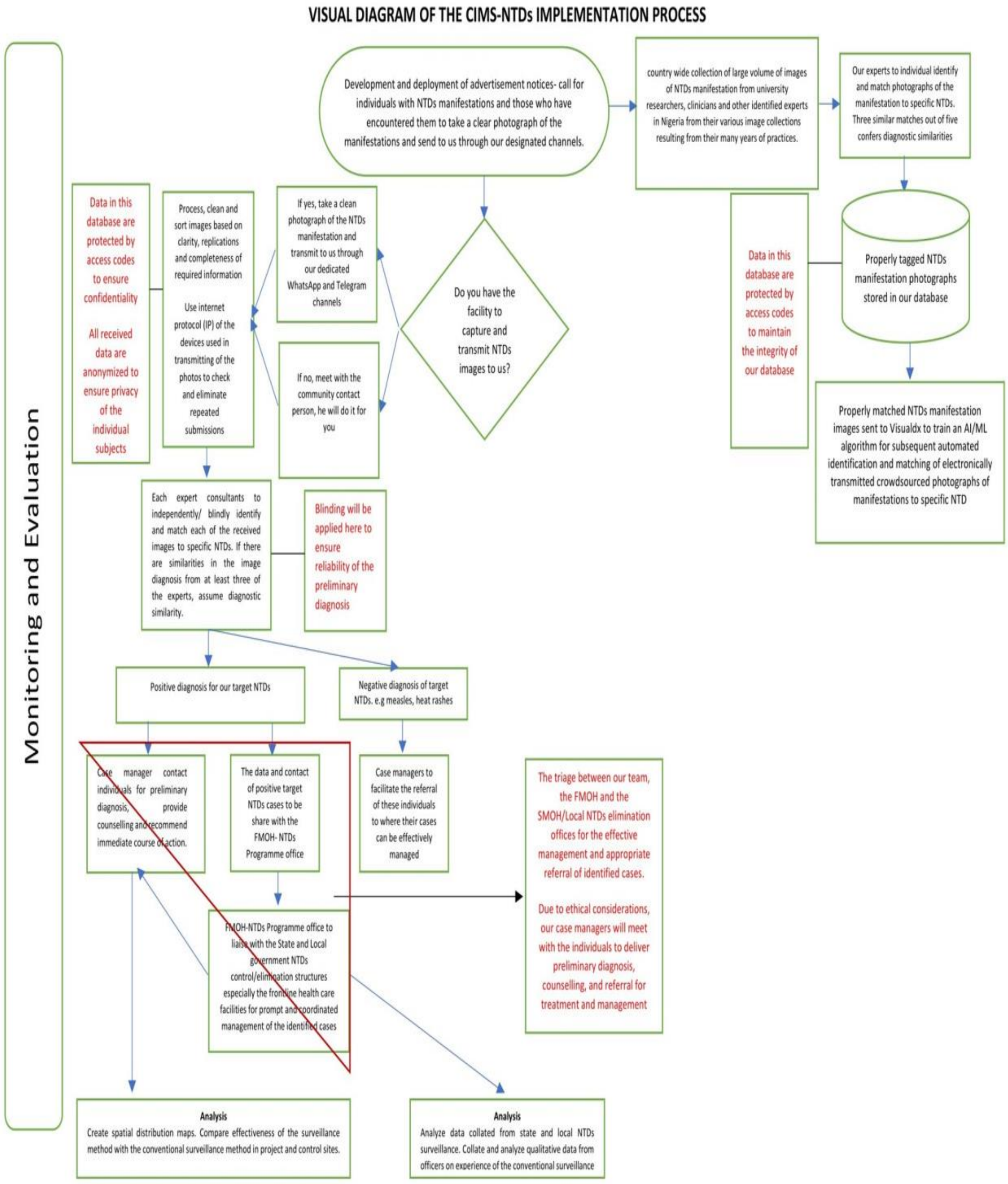

Figure 1: Flow diagram of the CIMD-NTDs process

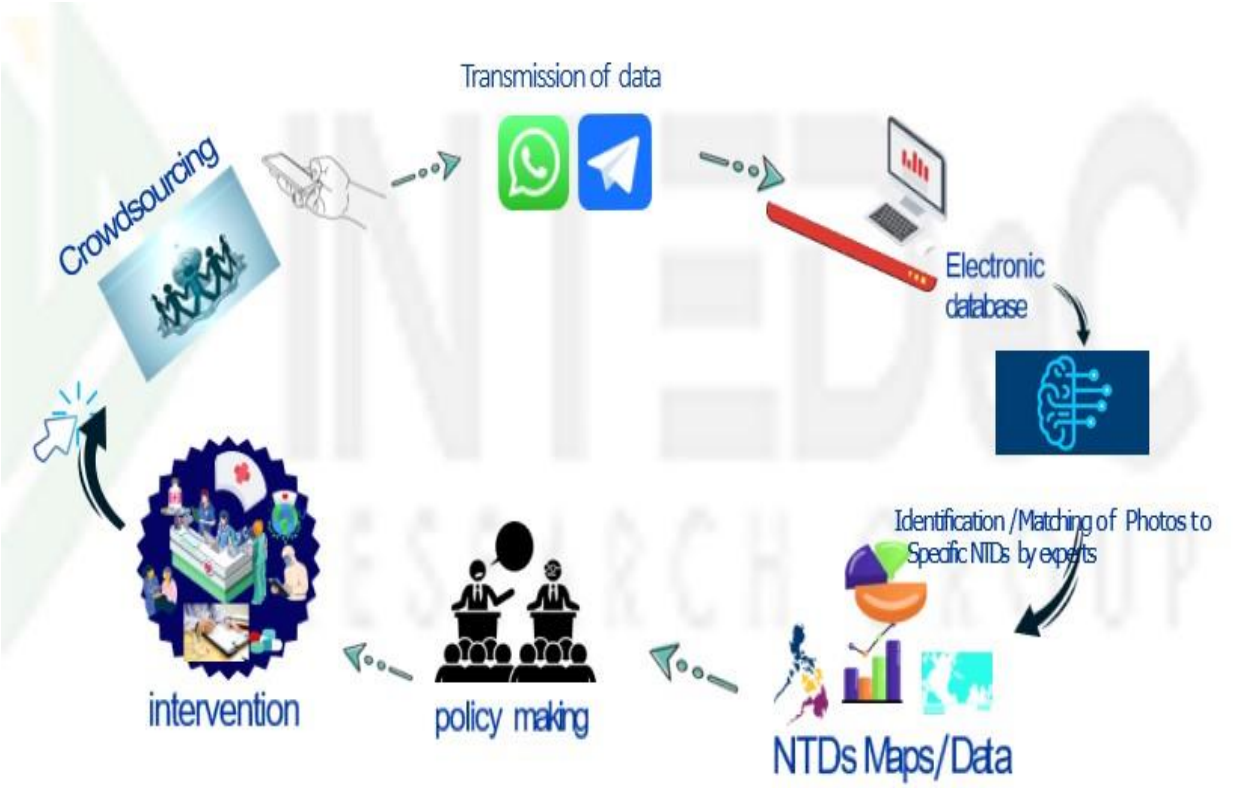

Figure 2: The simple pictorial depiction of the CIMS-NTDs process

Click here to access/download
Supporting Information - Compressed/ZIP File Archive
Supporting Information.zip
