# Supplementary material for: Protocol for the crowdsourced image-based morbidity hotspot surveillance for neglected tropical diseases (CIMS-NTDs)
Source: PLoS One. 2024 May 10;19(5):e0303179. doi: 10.1371/journal.pone.0303179 (PMC11086871; doi:10.1371/journal.pone.0303179)
Supplement: S1 File — (ZIP) [file pone.0303179.s001.zip › Proof of External Peer Review by Funders _INV-048721_08-22-2022 (1).docx]

**INVESTMENT DOCUMENT – GRANT**

**Cover Page & Document Guidance**

Welcome! The Investment Document is both your proposal shaping document, as well as your reporting document should your project receive funding. Before beginning, discuss the requirements for this document with your foundation contact for guidance and expectations on content, length, and level of detail.

| ****Proposal Sections**** | ****Reporting Sections**** |
| --- | --- |
| **Section A**constitutes the grant proposal narrative. Typically, this section and the budget template (separate Excel document) are considered your proposal package. | **Section B**contains progress narrative questions to be completed for each progress reporting cycle defined in your grant agreement.  **Section C** contains final narrative questions to be completed at the end of your grant. |
| Important Guidance:   - This document is not a commitment by the foundation to fund the work.**If a grant agreement is executed, the grant proposal narrative (Section A) and budget will be incorporated therein by reference.** - To better understand the restrictions on foundation funding, please review the foundation's [standard grant terms and conditions](https://docs.gatesfoundation.org/Documents/Sample-Terms-and-Conditions.pdf)*.* This is not an exhaustive list and is subject to change. This list is provided for informational purposes only and does not imply an award, agreement, or offer to contract. - This document must be submitted in English and as a Microsoft Word file. **PDFs and other file types will not be accepted**. - **Privacy and Non-Confidentiality Notice:** The Internal Revenue Service (IRS) administers U.S. federal tax laws and requires that the foundation publish a list of its grants. We may also provide a general description of our grants and contracts on our web sites, in press releases, and in other marketing materials. Subject to the foundation’s [Privacy & Cookies Notice](https://www.gatesfoundation.org/privacy-and-cookies-notice), the foundation may also share information you provide to us (either orally or in writing) with third parties, including external reviewers, key partners and co-funders. This document is subject to the foundation’s [Terms of Use](https://www.gatesfoundation.org/Terms-of-Use).   If you have any questions or need support, please reach out to your foundation contact at the email listed on the next page. | |

**Table of Contents**

Use the hyperlinked sections below to navigate the document:

| [SECTION A – GRANT PROPOSAL 3](#_Toc71535245)  [Investment Details 3](#_Toc71535246)  [Budget Information 8](#_Toc71535247)  [Roles and Responsibilities 12](#_Toc71535248)  [SECTION B – PROGRESS NARRATIVE(S) 15](#_Toc71535249)  [Progress Reporting Period 1 15](#_Toc71535250)  [SECTION C – FINAL NARRATIVE 20](#_Toc71535251) |  |
| --- | --- |

# SECTION A – GRANT PROPOSAL

| Investment Name | Crowdsourced Image-based Morbidity Hotspot Surveillance for Neglected Tropical Diseases in Nigeria | | |
| --- | --- | --- | --- |
| Investment ID | INV-048721 | | |
| Investment Owner | Lungi Okoko | Investment Owner Email | lungi.okoko@gatesfoundation.org |
| Investment Coordinator | Jennifer Cornelison | Investment Coordinator Email | jennifer.cornelison@gatesfoundation.org |

## Investment Details

| Estimated Grant Start Date | 01 November 2022 | Grant End Date | 31April 2024 |
| --- | --- | --- | --- |
| Requested Grant Amount (USD) | $ 299,928 | Total Project Cost (USD) | $299,928 |
| Organization Legal Name^[1]^ | Federal University of Technology Owerri (FUTO) |  |  |
| Organization Doing Business As/Trade Name^[2]^ |  |  |  |
| Fiscal Agent^[3]^(if applicable) | Not applicable | Fiscal Agent Contact Name (if applicable) |  |
| Tax Status (if known and applicable) [Refer to Tax Status Definitions](http://www.gatesfoundation.org/How-We-Work/General-Information/Tax-Status-Definitions) | Tax exempted | U.S. Employer Identification Number (EIN) (if applicable) |  |
| Mailing Address PMB 1526, Owerri, Imo State, Nigeria | | Primary Contact Name | Uchechukwu Chukwuocha |
| Street Address 1 |  | Primary Contact Title | Dr |
| Street Address 2 |  | Primary Contact Email | uchukwuocha@gmail.com, uche.chukwuocha@futo.edu.ng |
| Street Address 3 |  | Primary Contact Phone | +2348034712957 |
| City | Owerri | Additional Contact | Chinebuli Uzondu |
| State / Province | Imo State | Additional Contact Title | Dr |
| Zip / Postal Code | 460107 | Additional Contact Email | chinebuli.uzondu@futo.edu.ng |
| Country | Nigeria | Authorized Signer Name | Emeka Oguzie |
| Website (if applicable) | www.futo.edu.ng | Authorized Signer Title | Professor/ Deputy Vice-Chancellor (Research, Development and Innovations) |
|  | | Authorized Signer Email | emeka.oguzie@futo.edu.ng |
| Organization's Fiscal Year End | September | Organization’s Total Revenue for Most Recent Fiscal Year (USD) | $25,411,741.34 |
| Proposal Submitted By (name) | Uchecchukwu Chukwuocha | Proposal Submitted Date | 01 August 2022 |

### Charitable Purpose - completed by the foundation

- The following describes the charitable purpose of this project. It is written in a standard format by your foundation contact for publication on the foundation’s public website should your project receive funding.

To test the implementation of crowd sourced image-based disease surveillance for Neglected Tropical Diseases in Nigeria

### Strategic Fit - completed by the foundation

- The following includes the foundation’s 3-5-year strategic goal(s) towards which this project contributes most directly.

By 2030, we aim to equip all endemic countries in Africa with the tools and approaches needed to eliminate the morbidity caused by onchocerciasis, LF, STH, and SCH, and to sustain their gains in the long-term. This investment contributes to this goal by increasing access to real-time data on morbidity hotspots to identify subnational areas where more targeted, integrated surveillance and response efforts are needed. This investment will also expand equitable access to accurate diagnosis, referral, and care for NTD patients, including Lymphatic Filariasis, Onchocerciasis, and Female Genital Schistosomiasis.

### Project Description

*Note: this section may be pre-populated with your foundation contact's initial draft, please expand upon and/or continue to refine.*

In this section, please describe the proposed work, including a clear outline of the project plan, intended results, timeframe, funding considerations and how you and your organization will work to further educational, scientific, or other charitable purposes and impacts for the intended beneficiaries. Please be sure that your project description is sufficiently detailed and includes responses to the below questions:

- What are the primary results (outcomes and outputs) this project will achieve or significantly contribute to? How will you know when the results have been achieved? How will the results be measured?
- How will your management of the project and the intended outcomes align with the Charitable Purpose, Strategic Fit, and the foundation’s Global Access requirements?
- Describe potential risks/challenges to the success of this project and how you plan to address them. Include any external factors or critical relationships with other partners/projects that may influence the success of this project (including any anticipated agreements to be entered into for purposes of the project).
- Describe any changes or improvements you plan to make to your organization's capacity to achieve the results of the project.
- Beyond this project’s time frame and funding, what factors (financial/economic, organizational, behavioral, etc.) are required to sustain or build on the project’s results?

***Background***

Neglected Tropical Diseases (NTDs) have been classified with goals of elimination by the World Health Organization (WHO) in the NTDs Road Map 2021-2030 [1]. Recently (in 2022), the Kigali declaration on NTDs was madeto mobilize political will and secure commitments to achieve the Sustainable Development Goal 3 (SDG3:Good Health and Well-being) target on NTDs and to deliver the targets set out in the World Health Organization’s Neglected Tropical Disease Roadmap[2].Effective surveillance and targeted deployment of interventions are essential components of strategies towards the elimination of NTDs. Traditional surveillance systems are however time-consuming, expensive, and do not cover the entire population. Additionally, they are ineffective in conditions that prevent access to locations where NTDs may be endemic, such as the case of the COVID-19 pandemic, civil upheaval, and natural catastrophes. Surveillance based on crowdsourced data has been shown to provide an affordable, realtime, scalable and effective method to improve malaria surveillance in the recent past [3]. Based on the lessons drawn, the proposed project seeks to contribute towards achieving the WHO target for the elimination of NTDs by developing a crowdsourced image-based morbidity surveillance method to improve the surveillance of NTDs. This will be achieved through the following objectives:(1) Development and validation of an electronically transmitted crowdsourced photograph (remote) method to improve thesurveillance of NTDs - This involves sensitizing affected subjects or those around them in their communities to take photographs of the NTD manifestation upon notice with a smart device and transmit together with relevant demographics to a designated telephone number through social media handles, from where they are collated in an electronic data base, identified, and matched to specific NTDs by experts.  This together with location data will be used to map the specific NTDs as well as to determine their demographic and geographic distributions. This data can then be used by policy makers for better planning and targeting of interventions as well as enable improvements in timely appropriate case management. This process could also aid post elimination surveillance. This is because community members would have been sensitized and conscious to the point that once they experience or notice anyone in their communities with manifestations that look like those of the NTDs, all they will do would be to take a photograph of it and transmit along with relevant demographics to designated post elimination surveillance officials who will then take appropriate action such as identifying residual cases and missed communities as well as deployment available interventions. This would become a passive method of constant reporting of cases that may emanate after the elimination of the respective NTDs. (2) Development and validation of a feedback mechanism and personalized/individual care for NTDs subjects - With the data base of telephone numbers collated through the transmission of photos, subjects can be personally sent feedbacks on preliminary diagnosis of their conditions and appropriate immediate actions to take for morbidity management and disability prevention. Supportive non-clinical care such as counselling on hygiene practices and avoidance of transmission enabling behaviours . They can also be communicated through this medium on when, where and how to receive available interventions.(3) Development and training of an Artificial Intelligence (AI)/ Machine Learning (ML)algorithm for eventual identification and matching of received images to specific NTDs as well as preparatory to the development of a point of care Artificial Intelligence (AI) driven mobile application for differential diagnosis and management of NTDs at health facility levels - This will be done in partnership with VisualDx (an Artificial Intelligence company which uses visualization to support clinical diagnosis) and will involve using the data base of collated, identified and matched photographs coupled with knowledge data base of respective NTDs to create an AI driven matcher and mobile application, which could be deployed in health facilities. The mobile application will be designed to aid health personnel to differentially diagnose those presenting with NTDs manifestations in the health facilities as well as provide clinical management or referral advice in real time. However, the field testing of the point of care AI mobile application will not occur as part of this project.

It is expected that this phase of the project will provide a timely, less expensive, population inclusive, feasible, acceptable and more effective method as compared to the traditional/ conventional method of surveillance of NTDs. This project has potentials for scale up towards integration into ongoing plans and programmes for NTDs elimination and into the health system, more timely and less expensive large scale surveillance of NTDs across the country and sub-Saharan African region, surveillance of multiple NTDs at the same time and through one process, better diagnosis, case management and prevention, and ulimately contribution towards the elimination of NTDs by the set target for elimination. Furthermore, this method will be valuable for post elimination surveillance of NTDs so as to identify residual cases or missed areas and groups, and promptly deal with them to prevent resurgence. Another area that will be explored in the future will be the possible full automation and digitalization of the entire image-based surveillance and response process with very minimal human interference using artificial intelligence, machine learning and cloud computing tools, and even make it more personalized for the affected subjects.

***Methodology/Approach***

***Project Location:*** The project will be carried out in Nigeria. Nigeria is located in West Africa, and between latitudes 4 and 14 degrees north and 2 and 15 degrees east, sharing a border with Benin, Niger, Chad and Cameroon. The country has a population of over 216.7 million people with a population density of 226 per Km^2^. Nigeria has 36 states which are grouped into 6 geopolitical zones which are North-East, South-East, South-South, South-West, North-West and North-Central. It has a range of natural environments, from semi-deserts in the north to tropical rainforest in the south. Nigeria has a tropical climate with variable rainy and dry seasons, depending on location. It is hot and wet most of the year in the southeast but dry in the southwest and farther inland. Nigeria is one of the most densely populated nations in Africa, the most populous country in the African continent, and the seventh-most populous nation in the world. Hence, Nigeria is a likely target market for mobile phone, internet, and telecommunication businesses, and as such has influenced many Nigerians to becoming smartphones owners. It is estimated that about 90% of the country's population are mobile phone owners[4]. Nigeria has a significant amount of cultural diversity, due to its more than 250 distinct ethnic groups, more than 500 languages, and the variety of traditions practiced by each of them. Nigeria's economic situation is such that more than 40% of the population lives in poverty, with poor social and healthcare infrastructure. This situation together with environmental and climatic factors encourage the spread of a variety of diseases, including neglected tropical diseases. Nigeria bears the second highest global burden of NTDs.

***Inception Meeting of Project Partners(Month 1):*** This project will commence with a hybrid meeting (virtual and in-person)of all project partners.The project partners include the Federal Ministry of Health of Nigeria Programme Offices (NPO) for NTDs, VisualDX and NGOs such as Centerfor Integrated Health Program (CIHP), Female Genital Schistosomiasis Network (FGSN) Nigeria )-whose role will be majorly community mobilization, sensitization and follow up. This meeting will involve further familiarization of project partners, assessment of the project design, procedures and protocols, confirmation of assignment of roles as well as setting up the work flow / system for the project.

***Selection and Training of Research Assistants (Month 1):*** Three (3) post-graduate students of the department of Public Health, FUTO will be selected and trained to serve as assistants in the project.

***Design of Advertisement and Selection of Project Sites/Locations (Month 2):*** Photo and literature search will be done to select photos of manifestations of NTDs of focus (Urinary schistosomiasis, Onchocerciasis, Lymphatic filariasis, Trachoma, Buruli Ulcer, Guinea worm and Ascariasis). Selected photos would be validated by NPO officials using WHO standard operating procedures (SOP), and used to design the study advertisement/notice with content as follows: 1) Distinctive photos of the manifestations of the focus NTDs with descriptions in English and local languages of the respective study locations; 2) Call for individuals with NTDs manifestations or those who have encountered them to take clean photographs of the manifestation (with the consent of the subject and excluding their faces) using a smart phone, and transmit through Whatsapp or Telegram to phone numbers indicated on the advert, accompanied with demographics of the subject (location, age, gender, occupation, marital status, educational level), or to use a designated community contact person whose name and phone number are on the advert to do so on their behalf in case of lack of a device to carry out the activity; 3) Notice that the NPO would provide available intervention measures to those who comply with the request. Advert narratives will be in both English language and the respective local languages of the pilot locations.

Six pilot states will be purposively selected based on available data from the Federal Ministry of Health, Nigeria on the endemicity of the target NTDs and to cover the six geopolitical regions of the country. Three of these states will be randomly selected as the intervention states while the other three states will serve as controls. An additional criterion for selection will be, endemic states where the Partner NGOs are already operating. NTDs surveillance data will be collected from State NTD programme offices of both the intervention and control states. These data will be used to compare outcomes between our method and the conventional method within the intervention states as well as with the control states. The indicators to be used for comparisons include but not limited to proportion of suspected NTD cases (per disease) reported weekly/ monthly, rate/ timeliness of reporting of suspected cases, proportion of identified cases that received some response in the intervention states compared to the control states, promptness of the response received, demographic and geographic distributions of identified cases per focus NTD.

It is worthy to note that Female Genital Schistosomiasis (FGS) will not be part of the NTDs of focus in the remote surveillance aspect of this project because it does not manifest externally like others but internally, and therefore the image of manifestation cannot be easily captured with mobile devices by subjects.

***Advocacy Visits, Engagement of Local Stakeholders and Inception Workshop (Month 3):*** Stakeholders in the selected states including officials of State and Local Government Health authorities, religious and community leaders as well as local and international NGOs supporting NTDs programmes in both the intervention and control states will be engaged to solicit their support in sensitizing/ mobilizing community members and in any other way that could help the project to succeed.Community contact persons will be selected among Community Directed Distributors of ivermectin or Community Health Workers and trained for this work. An inception workshop will be held to interact with and seek the opinion of the key stakeholders on the purpose and procedures of the project which will be vital in its execution.

***Intervention Communities' Sensitization and Mobilization (Month 4):*** Sensitization and mobilization of the communities in the intervention states will be carried out soon after the stakeholder engagement. This will be led by the partner Non-Governmental Organizations (NGOs) with the support of the local stakeholders. This is aimed at creating awareness about the project among community members and carrying them along through the Will these one off initial engagement activities be complemented with some on-going engagement through out the project to foster community ownership, and allow adaptation for optimization of project implementation?. Beyond this, there will be periodic advocacy visits to the project communities and continous engagement with the community stakeholders physically and virtually to carry them along althrough the project in order to foster community ownership.

***Production and Deployment of Project Advertisements/Notices to Intervention States/ Sites (Month 4):*** Advertisements/notices that have been designed will be produced after validation by the Federal Ministry of Health's national NTD programme office and deployed in intervention states as follows:(1) Billboards- to be deployed at strategic locations such as community centers, (2) Posters- to be distributed at primary, secondary and tertiary health facilities, commuter parks, religious centers, markets and schools, (3) Handbills- to be distributed at market squares and other public places. Additional adverts/notices will be placed on local TV, Radio stations as well as social and print media. These are aimed at adequately sensitizing the public on the procedures and their involvement in the process. Control states will continue with the traditional surveillance systems without the intervention.

***Large Volume Collection of Photographs of Manifestations of NTDs and Development of Artificial Intelliegence (AI) driven Matcher of Images to Specific NTDs (Month 4-7):***

At the early stage of the project, expert consultants with extensive experience in NTDs research together with NPO officials from the Federal Ministry of Health and personel of the partner NGOs will amass, validate, and match a large volume of photographs of different stages of the manifestations of NTDs prevalent in the project locations using digital cameras. Additionally, researchers from universities, clinicians and other identified NTDs experts in Nigeria and other parts of the World will be engaged to seek their contribution of photographs of confirmed NTDs cases from their various image collections. Photographs collected through these processes will be used to generate the data set needed to create and train the Artificial Intelligence (AI)/ Machine Learning (ML) algorithm for subsequent automated identification and matching of electronically transmitted crowdsourced photographs of manifestations to specific NTDs. These data set together with accompanying knowledge data base will also form the basis for the development of the point of care differential diagnosis and management mobile application. The algorithm will be tested on test set of images as well as comparison with the outcome of the expert matching process to confirm validity and  diagnostic accuracy prior to its application on crowdsourced images. The development of the AI/ ML photo matching algorithm and point of care mobile application will be led byVisualDx **(**an Artificial Intelligence companywhich uses visualization to support clinical diagnosis).

Furthermore, this project will involve five experts who will identify and match received images of NTDs. Each of these experts will work blindly on the same images. If there are similarities in the image diagnosis from atleast three of the experts, we will assume diagnostic similarity. This will also serve as a means of quality check among the experts.

***Reception, Collation, Processing and Analysis of Photo and Demographic Data, and other Data (Month 7-15):*** Transmitted photos and demographic data will be received and collated in a central electronic data base and protected with an access code. Photos will be processed and sorted based on clarity, replications and complete data. Internet Protocol (IP) of the devices used in the transmission of the photos will be checked to eliminate repeated submissions. All data will be anonymized so that they will not be linked to individuals. A data protection expert will be involved to ensure compliance with relevant data protection laws. Initially, we will work with our expert consultants to identify, confirm, and match crowdsourced images to specific NTDs, while work on development, training and validation of the AI/ ML algorithm is ongoing.As soon as the AI/ ML algorithm has been trained and validated to the extent of confidence required to screen and matchtransmitted images, it will then be applied to replace identification/ matching of images by expert consultants. Subsequently, therefore, the AI/MLwill automatically identify and match crowdsourced photos tospecific NTDs.Arc Map, a digital mapping software will thereafter be used to map the respective target NTDs using the identified/ matched photos together with the location data along with the GPS location imprints of the transmitted images. Demographic and geographic data will be analysed using statistical software packages like SPSS and R to determine geographic and demographic distribution of these NTDs. Towards the end of the photo or image data collection, quantitative and qualitative techniques will be use to collect data from the subjects on their experience, acceptability, usability and feasibility of this crowdsourced image-based NTDs surveillance method. Data will also be collected from officers at local government NTDs programme offices the on their experiences with the conventional NTDs surveillance method and possible perceptions about this new method.

***Feedback on Preliminary Diagnosis and Information on actions to take (Personalized/ Individual Care) (Month 7-15):*** Concurrently with the period of data transmission, collation and processing, there will be feedback to subjects whose data were transmitted through the telephone numbers used in transmitting such data. This will be done as the respective photos are successfully matched to specific NTDs. The feedback will be on the preliminary diagnosis of their conditions, counselling, and provision of information on appropriate actions to take as well as follow up, which will be undertaken by the project case managers and officers who are physicians with experiences regarding. They will also visit the communities periodically to provide diagnosis physically and support to identified subjects NTDs. Together with the FMOH's national NTD programme and local authorities/ health facilities as well as the community contact persons, standard operating procedure (SOPs) for investigating, triaging between the Federal, State, Local Government NTD control/ elimination programme offices and responding to NTD cases that are confirmed remotely will be developed to ensure confirmatory diagnosis and appropriate treatment is provided. This will involve leveraging on existing NTDs control/ elimination structures which includes frontline health facilities in every Local Government where identified cases of NTDs will be managed and counselled, and referal to tertiary health facilities where more serious cases beyond the capacity of the frontline facilities will be handled. Community contact persons together with selected community health workers at the local or community levels will additionally be trained as adherence counselors to track individuals who transmitted images and received feedback to ensure that they take recommended actions and assist in delivering specific messages against stigmatization.

***Development and Training of the Artificial Intelligence/ Machine Learning Platform for the Identification and Matching of Transmitted Images to Specific NTDs (Month 7-15):*** Artificial Intelligence (AI)/ Machine Learning (ML) training and iterative development/research tasks will be pursued to support development of an NTD targeted module by VisualDx working in collaboration with Nigerian partners, who will aid in sourcing images and providing additional content expertise. A deep neural network will be trained to predict select NTDs including Dracunculiasis, Buruli Ulcer, Visceral leishmaniasis and PKDL, Leprosy, Lymphatic filariasis and Onchocerciasis along with their clinical mimics. NTD images will be part sourced from Nigeria through in-country consultants to lead image collection and curation along with other Nigerian NTD experts, faculty members of universities and clinicians who are members of the Parasitology and Public Health Society of Nigeria. Additional clinical images will be sourced from open datasets, solicited images and cases from clinicians and international professional societies. Images for other diagnoses, clinical mimics of the selected NTDs, will be pulled from VisualDx proprietary database of 100,000’s of clinical images. Concurrently the full list of disease represented in VisualDx will be paired down to those pertinent to the differential diagnoses of the selected NTDs. Working with a panel of experts and in collaboration with Nigerian colleagues, clinical questionnaires will be created to be incorporated into our VisualDx NTD module that will collect information about signs and symptoms pertinent to NTD presentations as well as to their clinical mimics. Using these image collections, we will incorporate them into existing VisualDx ML algorithms and conduct a series of tests and iterative enhancements for each included NTD to demonstrate accuracy and the utility of model (internal validation). We will also enable collection of demographic and location data associated with instances of positive NTD syndrome identification to enable public health syndromic surveillance (tracking cases over time, region, and social determinants of health).

Our initial metrics to assess accuracy of the AI/ML algorithm will involve iteratively evaluating it against a test set of unknowns which will include images not only of the select NTDs, but of their clinical mimics as well as other common acute skin complaints.

***Dissemination Seminars, Drafting and Submission of Final Report (Month 16-18):*** A dissemination seminars will be carried out at the end of the project to inform all stakeholders of the outcome and implication of the project. A report will be drafted and submitted to the Bill and Melinda Gates Foundation, the Federal Ministry of Health of Nigeria, as well as State and Local Ministries of health and other local stakeholders.

**Ethical Considerations and Informed Consent**

Ethical and protocol approvals for this project will be obtained from the Federal Ministry of Health, Abuja, Nigeria. Ethical clearance and approval will also be obtained from the research and ethics committee of the School of Health Technology, Federal University of Technology, Owerri, Nigeria as well as the Institutional Review Board of the Institution respectively.

Our advertisements will bear a brief description of the project, its intentions and the role of potential participants in lay language, and request that only those who give consent to participate should transmit images of NTDs manifestations (without their faces) and accompanying demographics (without their names). To further validate informed consent, there will be an automated response from our whatsapp and telegram platforms propmting those who transmit images/ data to confirm their consent and permission to use their data in this research. Additionally, our community contact persons who will assist those without smart devices to take photographs of the images and transmit will collect informed written consent from such subjects. For the children and vulnerable groups, their parents and care givers will be allowed to provide the consent of their behalf with the children assenting to it. Hard copies of signed consent forms will be collected from our Community Contact Persons and stored in a secure steel cabinet while electronic consent will be stored in our password secured elctronic data base.

**Intended Results**

It is expected that this project will deliver a novel remote surveillance and differential diagnosis/ management system which will significantly contribute towards achieving the elimination target for NTDs. Specifically, the project is expected to generate an inexpensive, real-time and population inclusive method for the surveillance of NTDs, a feedback mechanism for personalized care for NTDs subjects, a protocol for the electronically transmitted crowdsourced photographs and feedback mechanism method for the surveillance of NTDs, large crowdsourced photo data base of manifestations of NTDs, data on demographic and geographic distribution of NTDs, digital map of NTDs generated from crowdsourced photographs and demographic data, data from the subjects on their experience, acceptability, usability and feasibility of the crowdsourced image-based NTDs surveillance method, AI driven algorithm for automated identification and matching of NTDs manifestations to specific NTDs and datasets for the development of a point of care AI driven differential diagnosis and management mobile application . Furthermore, the project will help in improving awareness about NTDs and their mode of transmission, recognition, prevention, management and control.

The results will have been achieved when:(1) A protocol for the electronically transmitted crowdsourced photographs and feedback mechanism method for the surveillance of NTDs has been established. (2) The subjects or those that encounter them in the communities are able to take photographs of the NTDs manifestations upon notice using smart phones/ devices and transmitsame with necessary demographicsto designated phone numbers through social media channels for onward processing, feedback, planning and targeted deployment of appropriate interventions.(3) Crowdsourced photo data base of manifestations of NTDs as well as data on demographic and geographic distribution of NTDs across the project locations have been set up.(4) Transmitted photos of NTDs manifestations are effectively identified and matched to specific NTDs. (5) Digital maps as well as demographic/ geographic distribution data of respective target NTDs across the project locations have been successfully produced. (6) A feedback mechanism on initial diagnosis and advise on appropriate actions to take has been successfully established (7) AI/ML driven algorithm for the identification and matching of photos with specific NTDs has been created and trained.

The management of this project and its intended outcomes will enable population inclusion or community involvement in the surveillance of NTDs and by extension processes towards the elimination of NTDs. It will enable real-time/ rapid and inexpensive identification of groups and/ or areas where NTDs are prevalent without restriction of access to such areas by factors such as unrests, conflicts, poor road conditions and transportationas well as other situations such as that created by the COVID-19 restrictions. It will also enable the identification of missed subgroups (i.e., subgroups that were missed during routine surveillance). It will allow feedback as well as personalized care to individual subjects. It will aid in the better planning and targeting of interventions as well as in post elimination surveillance. It will also enable appropriate facility level differential diagnosis and management of NTDs in hard-to-reach areas.

**Monitoring and Evaluation**

The project will be monitored monthly till its conclusion. Checks and balances will be performed through project implementation meetings conducted at the end of every month. This meeting will involve all project partners as well as members drawn from the Federal University of Technology, Owerri, Nigeria Centre for Research, Innovations and Development (CRID). FUTO CRID has the responsibility to evaluate and monitor research activities domiciled in the university. The centre has existing protocols for doing this. At each meeting, the extent of achievement of the project objectives will be accessed via established indicators such as number of images transmitted, proportion of images successfully identified and matched to specific NTDs, number of subjects receiving preliminary diagnosis via feedback mechanism established, number of subjects who took action, percent increase in incident cases, accuracy of AI model for disease detectionetc. There will be a monitoring and evaluation (M&E) officer in the project team to ensure that set objectives are achieved within the stipulated budget and timeline.Progress report will be dulysubmitted to BMGF as required.

Intervention outcomes such as the proportion of suspected NTD cases (per disease) reported by state and local health authorities through existing weekly / montly surveillance systems reports in the control sites compared with visually diagnosed NTD cases reported, determination of demographic and geographic distribution of respective focus NTDs in the intervention and control states, as well as rate/ timeliness of response to identified individual cases or communities by designated health authorities in the intervention and control states and propoortion of identified cases and communities receiving feedbacks and appropriate response/ interventions.

**Potential Risks and Challenges**

The protocol and procedure for this project to a reasonable extent provides a risk mitigation strategy, considering that there will be no frequent interaction with individuals and communities during the project. However, when such interactions arise, there may be risk of exposure to transmission of endemic NTDs and other public health emergencies such as COVID-19, monkey pox. This will be mitigated by adhering strictly to prevention protocols as stipulated by the World Health Organization (WHO) and the Federal Ministry of Health of Nigeria guidelines. Any risk of exposure of subjects’identity would be mitigated by anonymization, which means that the NTDs manifestation images and accompanying demographics of the subjects will strictly not be transmitted with their faces and names. Subjects will only be identified by numbers. Risk of unauthorized access as well as loss of data will be mitigated by storing all data in a secured and encrypted folder.The potential risk of not having subjects submit images because they are not aware of the program or are afraid to submit will be mitigated through our extensive sensitization activities through various media such as posters, billboards, handbills, radio, religious centers and community mobilization which will involve trusted community stakeholders/ leaders and local NGOs. Potential challenges related to having someone, who received feedback on action to take after submitting a photo, not being able to take the recommended action of going to the health facility will be addressed by having the community contact persons to follow up as adherence counselors on these individuals to ensure that they take recommended action. They will serve as liaison to report any challenges to the local health authorities or project office who would make efforts to resolve any reported challenges. They will always be available in their communities to ensure that recommended actions are taken by subjects.. The risk of stigmatization of subjects will be mitigated through continous community awareness and sensitization on the NTDs with generic messages in patnership with the community stakeholders such as the community leaders, religious leaders, patent medicine vendors, community health workers and NGOs to reduce the level of misinformation, misconceptions and myths sorrounding the NTDs, which are the drivers of stigmatization. These sensitizations will also highlight the negative implications of stigmatization to the community and its members. Peer support groups will be created by the community contact persons in the communities where subjects encourage themselves to get involved in disease control activities such as ours and other community based activities. Furthermore, we will create dedicated helplines through which concerned subjects can reach our case managers/ counsellors for counselling and to address any of their concerns.

**Improvement to Organization's Capacity**

Considering that our organization is a higher education research institution, knowledge will be passed to students by getting them involved in aspects of the project. Potential postgraduate students will be encouraged to take up research projects in this area in order to ensure sustainability of this very important project. Partnership and collaborations with other relevant disciplines in the university and organizations outside the university will be established to ensure multidisciplinary approach as well as success and sustainability of the project. The experiences and expertise acquired by members of the project team drawn from different disciplines in our institution will be translated to other colleagues and students, and will help improve interdisciplinary as well as implementation research capacity of our institution. Methods and results from this project will contribute to research informed teaching in our institution, The success of this grant will as well enhance our organization's confidence in seeking for more international research grants and the confidence of granting organizations to award more research grants to our institution in the future.

**Factors Required to Sustain and Build on Project Results**

Remote surveillance and differential diagnosis of NTDs is a novel method that will contribute significantly to the WHO target for NTDs elimination and should be sustained beyond the project timeframe and funding. However, this would depend on factors related to integration of project protocols and procedures into ongoing plans and strategies of the Federal Ministry of Health of Nigeria and the WHO towards the elimination of NTDs. This will ensure coverage with budgetary allocations for NTDs elimination programmes, therebycontributing to project sustainability. Other factors include human and organizational capacity. There is need for continuous training and capacity building of human personnel and organizations that will be involved in implementing the project. Considering that the national funding allocations may not be adequate, there is a great need to attract external funding through grants particularly to scale up the project. Furthermore, there is need for local stakeholder support and community involvement to encourage the adoption of this novel method to improve surveillance aswell as the differential diagnosis and management of NTDs at community levels. There is need also for continued sensitization to deepen the consciousness of this method in the populationsuch that that once people experience any NTDs manifestations, they will just take a photograph of the manifestation with a smart device and transmit to designated authority phone numbers with the hope that they will receive feedback and appropriate interventions.

**References**

1. World Health Organization. Neglected Tropical Diseases, 2021. (accessed 29 November, 2021);	Available online: https://www.who.int/neglected _diseases/diseases/en/

2. Uniting to Combact Neglected Tropical Diseases Coalition. The Kigali Declaration, 2022. (accessed 11 June 2022); Available online: https://unitingtocombatntds.org/kigali-declaration/

3. Adekembi, O., Oyewusi, W.F., & Ogundepo, E. Rea-Time Crowdsourcing of Health Data in a Low-Income Country: A case Study of Human of Human Data Supply on Malaria First-Line Treatment Policy Tracking in Nigeria. In CWS@Neurips; 2020: 14-18

4. Gilbert P. Connect Africa: Nigerian Internet and Mobile Penetration Grows, 2021. (accessed 19 August 2022); Available online: https://www.connectafrica.com/author.asp

### Investment Results - Outcomes and Outputs

In this section, please provide specific details on the results this project will achieve.

**“Outcome”** is the ultimate or overall change(s) insystems, populations, or behaviors that a project seeks to achieve within the time frame of the grant.

- Describe what success looks like for the project by stating the intended outcome(s) of the project and how they relate to the activities funded by the grant.

The intended project outcomes and how they relate to activities funded by the grant are as follows:

1. Improvement in the surveillance of NTDs. This relates to the real time electronic transmission of crowdsourced images of NTDs to designated data base, where they are collated, processed, analysed and automatically matched to specific NTDs to develop maps of the respective NTDs and to determine their demographic and geographic distributions.

2. Improvement in community awareness of NTDs as well as prompt recognition of NTDs manifestations. This relates to the sensitization created in the course of this project particularly with the advertisements and notices.

3. Efficient feedback mechanism and personalized care. This relates to the collated telephone number data base of subjects or those around them, through which feedback and personalized care will be provided to individual subjects.

4. Facility level differential diagnosis and management of NTDs. This relates to the AI driven point of care mobile application for differential diagnosis and management of NTDs to be developed and trained for possible deployment at health facilities.

**“Output**” or “**Funded Development**” means the products, services, processes, technologies, materials, software, data, other innovations, and intellectual property (IP) resulting from the project (including modifications, improvements, and further developments to Background Technology).

- Please populate the table below with the intended outputs of the project. Add more rows as needed.

| Number | Output/Funded Development Description | Target Completion Date | Is a Third-Party agreement required? If yes, by when? | Will any IP rights be filed/generated?^*^ |
| --- | --- | --- | --- | --- |
| 1 | A curated list of other image-based crowdsourced surveillance in Africa and similar settings and how lessons will be incorporated in the protocol | 31 February 2023 | No | No |
| 2 | Protocol for remote surveillance of NTDs through electronic transmission of crowdsourced photographs of NTDs manifestations | 31 February 2023 | No | No |
| 3 | A list of messages advertising the system (promoting the benefits of submitting photos and what will be done with them) validated by the FMOH's national NTD program | 31 February 2023 | No | No |
| 4 | Photo data of different stages of NTDs manifestations | 31 April 2024 | No | No |
| 5 | Demographic and Geographic data of NTDs distribution | 31 April 2024 | No | No |
| 6 | Data on images of NTDs manifestaions phyically matched to specific NTDs | 31 April 2024 | No | No |
| 7 | Data from the subjects on their experience, acceptability, usability and feasibility of the new crowdsourced image-based NTDs surveillance method | 31 April 2024 | No | No |
| 8 | Data from local NTDs surveillance officers on their experiences with the conventional surveillance methods and possible perceptions about the new method | 31 April 2021 | No | No |
| 9 | AI driven matcher of electronically transmitted images of NTDs manifestations to specific NTDs | 31 April 2024 | No | Yes |
| 10 | Final Project Report | 31 April 2024 | N0 | No |

**Note: In future**progress report(s)* *you will be required to report on these outputs, and to disclose and update intellectual property (IP) and include any links to IP applications, filings, or registrations, as applicable.*

- If necessary, please add further explanations below:

### Global Access – Charitable Impact for Beneficiaries

To ensure a positive impact for the intended beneficiaries, the foundation requires that all projects and outputs be managed to ensure Global Access.

**“Global Access”**is a foundation policy requiring that: (a) the knowledge and information gained from the project be promptly and broadly disseminated; and (b) the Funded Developments be made available and accessible at an affordable price (i) to people most in need within developing countries, or (ii) in support of the U.S. educational system and public libraries, as applicable to the project. For more information and resources on Global Access, see the foundation’s [Global Access Statement](https://www.gatesfoundation.org/about/policies-and-resources/global-access-statement) and [Global Access webpage](https://globalaccess.gatesfoundation.org/).

- How will you disseminate the knowledge and information gained from the project? For additional information for peer-reviewed publications, see the foundation’s [Open Access policy.](https://www.gatesfoundation.org/How-We-Work/General-Information/Open-Access-Policy)

Dissemination of the knowledge and information gained from the project will be done through diverse mechanisms. First, there will be a dissemination seminar where projectknowledge and information will be communicated to the stakeholders. Second, policy briefs will be developed and served to decision makers for timely implementation and decision making. Third, findings will be made publicly available via scientific publications in open access peer reviewed journals and presentation at international scientific conferences. Fourth, project knowledge and information will also be made publicly available on our organizational website, Bill and Melinda Gates Foundation website as well as the websites of other partner organizations. Furthermore, the outcomes and outputs of the project will be posted on social media platforms such as Facebook, Twitter and Instagram.

**“Background Technology”**means all products, services, processes, technologies, materials, software, data, or other innovations, and intellectual property created by you or a third party prior to or outside of the project and used as part of the project.

**Please list any** **Background Technology****that will be used in the project.** Commonly available, off-the-shelf products (such as Microsoft Excel, Adobe, etc.) do not need to be disclosed.  Add more rows as needed.

*Note:* *Background Technology* *previously funded by the foundation will be considered a* *Funded Development* *for purposes of* *Global Access* *and any* [*license to the foundation*](https://docs.gatesfoundation.org/Documents/Humanitarian-License-Nonbinding-FAQ.pdf)*.*

| List each Background Technology to be used in the project | Is this Background Technology owned, controlled, or developed by you or sublicensable by you? | Do you need permission or a license from any third party to use this Background Technology to achieve Global Access? | If any permission/license is needed, please detail below, and describe your plan and timeline to obtain such permission/license or submit a copy of the agreement. | If this Background Technology is subject to Intellectual Property rights, please identify and include any links to applications, filings, or registrations, as applicable. |
| --- | --- | --- | --- | --- |
|  | No/Yes | No/Yes |  |  |
|  | No/Yes | No/Yes |  |  |
|  | No/Yes | No/Yes |  |  |

- How will you ensure affordable and meaningful access to the Funded Developments and Background Technology, if any?

Not applicable

- **Do you foresee any obstacles to achieving** Global Access (e.g., third-party rights, restrictions on Background Technology, time frame, affordability)?

__No_No___Yes (please explain and describe the specific steps that you will take to address the obstacles)

- For Global Access purposes, please confirm that you will make available the funded developments and any Background Technology that is (a) owned, controlled, or developed by you, or in-licensed with the right to sublicense; and (b) either incorporated into a funded development or reasonably required to use the funded development. For more information, see the Global Access terms located in the foundation’s [standard grant terms and conditions.](https://docs.gatesfoundation.org/Documents/Sample-Terms-and-Conditions.pdf)

___Confirmed___Not confirmed (please explain)

Not applicable

## Budget Information

The purpose of this section is to supplement the information provided in the Excel budget template by justifying how the budgeted items are necessary to implement project activities and accomplish project results. Please focus on key costs and risksand avoid repeating information contained in the budget template. Together, this narrative section and Excel budget template should provide a complete description that supports the proposed budget. For questions below that are not relevant to your project, answer “N/A”.

For additional instructions and guidance on the budget template, see the [Grant Budgeting & Financial Reporting Detailed Instructions](https://docs.gatesfoundation.org/documents/budget_template_instructions.docx).

### Budget Summary

- Explain the major cost drivers and how costs relate to planned activities and target outcomes. Also, explain any potential risks in spending and any plans to mitigate those risks. In the Excel budget template, an *additional dimension* can be defined to break down costs beyond the standard expense categories (e.g., by geography, product candidate, projects within a portfolio, or other). If you are defining such an additional dimension, please use it as you explain the major cost drivers here.

This budget was made to ensure efficient delivery of the proposed project as well as to realize the set objectives. To ensure wide coverage and awareness and sensitization, 9.7% of the budget will be subcontracted for the production and deployment of materials and activities for this purpose. Since this is a new innovation, this will enable full utilization of diﬀerent media in creating awareness, and sensitization on procedures that will require the involvement of members of the community in this project as well as on awareness about NTDs. About 30.8% allocated for personnel cost will be used to engage 12 full time personnel spanning diﬀerent specializations and work functions that will be responsible for the day- to- day activities and smooth running of the project from prior initiation to the completion of the project. Also, this cost will cover for stipend for 3 post graduate research students to be trained in this project. Other major cost drivers include travels (24.5%) which will involve onsite visits, advocacy calls and to address site specific cases from start to end of the program. Also, there will be a visit to the Federal Ministry of Health in Abuja for interfacing with the NPO for NTDs towardseasy adoption and continuation at the end of the project. This budget takes into cognizant the inflation rate and the high volatility of the Nigeria currency (naira) and as such all contract and reoccurring expenditure like procurement of items, transport fare, internet usage will be contracted and policy made to close all bidding at the initiation of the project.

### Detailed Budget Information

In this section, please complete the sub-sections that apply to your project and associated budget, and answer “N/A” for those that do not apply.

#### Personnel and Benefits

- Provide a description of budgeted personnel (including staff to-be-hired) and their responsibilities as they relate to the project. For to-be-hired individuals, please make sure your budgeting assumptions factor in realistic timing for recruiting.

Describe the components included in the benefits percentage (column R of the “Budget Details” sheet in the budget template). For example: pension, health insurance, expatriate costs, etc.

There will be one principal investigator (PI) and five co – investigators (Co-Is) whose responsibility will be the overall coordination and implementation of this project. These individuals have rich experience and expertise in the field of Public health, Parasitology, Data analysis project planning and implementation. There will be program manager, program officer, monitoring and evaluation officer, finance officer and case manager. The program manager will work hand in hand with all investigators to ensure that all activities and tasks are carried out to the latter. The program officer will assist the program manager to ensure easy flow of communication and distribution of responsibility. The monitoring and evaluation officer will ensure that project objectives and realized with time frame and budget. Finance officer will handle the finances of the project, including disbursement of funds, account reconciliations and retiring. Three case officers, one for each intervention state) who will be a medical doctors will evaluate and provide advices on cases of NTDs that require such during the project. There will be ~ 18% inflation rate according to Nigeria bureau of statistics (May, 2022). This inflation rate is factored into personnel’s wages to also accommodate its rise. About 10% and 5% travel benefit will be allocated to the PI and Co-Is and other personnel respectively to cover for travel bonuses as captured in the personnel cost

#### Travel

- Describe the travel budgeted and the assumptions used to estimate the appropriate number of trips and associated costs.

About 24.5% of the overall grant will be budgeted for travels. This will enable on- site visits for inspection, advocacy visits, addressing any issues and collaboration. Three intervention states will be selected for this project. Each state will be visited twice, subsequently; we will use virtual conferences and teleconference for monthly update. Because of the virtual conferencing and teleconference, two visits are enough for the project and using the projected inflation rate in the country, 24.5% allocation will be able to cover for transport, meal and accommodation for two personnel. There will also be a visit to VisualDx at Rochester, New York, USA.

#### Consultants

- Describe the work to be performed by consultants and any reimbursable expenses that have been budgeted in addition to consulting fees.

The cost in this category will cover for experts from the Federal Ministry of Health and researchers with extensive experience in NTDs research to help in onsite photo data collection and matching with specificNTDs which will be used to generate the data set needed to create the Artificial Intelligence (AI) algorithm.

#### Capital Equipment

- Describe any budgeted capital equipment items and explain why they are needed for the project. Only items with a unit cost of more than $5,000 (USD) and a "useful life" of more than one year should be included here, whereas items with a lower cost or shorter life should be budgeted under Other Direct Costs (see below).

N/A

#### Other Direct Costs

- Describe other items required for the project that fall under direct costs and are not part of any cost category above. For additional information on direct and indirect costs, please see the foundation's [Indirect Cost policy](https://docs.gatesfoundation.org/documents/indirect_cost_policy.pdf).

There will be purchase of office equipment such as; laptops, tablets, cloud storage, printers, photocopier (for monitoring, evaluation,) smart phones - (for data capturing and transmission), and motorbike fare for conveyance of volunteers at different communities in the pilot states.

### Subawards

Subawards are contracts or grants that you, as the prospective primary grantee, negotiate with other organizations who contribute to the completion of this project. A subaward is “under” the main grant award; as the primary grantee you will receive funds from the foundation and manage the disbursements to the sub awardee. You are responsible for negotiating the subaward budget, overseeing the work of the subawardee, disbursing funds in accordance with the subaward agreement and ensuring all applicable policies are met. For additional information, see [Exercising Expenditure Responsibility Over Subgrants](http://www.gatesfoundation.org/Documents/Expenditure%20Responsibility%20Procedures%20for%20Subgrants.docx).

**If your project will include subawards, please include the following information:**

If you are an Indian organization/entity subject to India’s Foreign Contribution Regulation Act (FCRA), then in compliance with FCRA, do you confirm that your proposed project will not include any subgrantees? This prohibition does not preclude subcontracts in the ordinary course of business.

_Not applicable__Not applicable     ___Confirmed     ___Not confirmed (please explain)

.

- In the Excel budget spreadsheet, please enter each subaward as a separate line item with the expected disbursement for each period broken out.

If your proposal includes any subawards greater than $1 million USD, please provide a separate budget spreadsheet for each subaward as an appendix to the proposal materials, using the foundations Excel budget template. Please reach out to your foundation contact if you need a blank budget template or have questions or concerns about using this format with any of your subawardees.

- In addition to the information included in the Excel budget template, please also list all subgrantees or subcontractors involved in this project in the table below. Add more rows as needed.

| Type of Subaward (grant or contract) | Organization Name | Organization Location(Country) | Website or Email  (please include email if subcontracting to an individual) |
| --- | --- | --- | --- |
| Production of billboards | to-be-determined | Nigeria |  |
| Production of posters | to-be-determined | Nigeria |  |
| Production of handbills (fliers) | to-be-determined | Nigeria |  |
| Radio adverts | to-be-determined | Nigeria |  |
| Newspaper adverts | to-be-determined | Nigeria |  |
| TV adverts | to-be-determined | Nigeria |  |
| Internet/Data | to-be-determined | Nigeria |  |

- In the space below, provide the following additional information for each organization chosen to participate on this project as a subawardee:

Describe the work the subawardee is going to perform, what selection process was used, how the sub-budgets were negotiated, and what the key cost drivers are. If organizations are not yet known or "to-be-determined" (TBD), include the assumptions used to estimate the cost for the subaward, and the process and timeline you will use to select these organizations and negotiate budgets. Once subaward organizations are selected, you will be required to submit the subaward information and budget (as necessary) to the foundation in writing.

| The subawardee will produce the advert/sensitization materials. Most of the reputable advertisement companies in the country have website where their service cost can be extracted and as such there will be a short timeline in negotiation and contracting to bidders. Because of the peculiarity of the project in which the success of the intervention lies on a wider coverage of publicity and awareness. Most of the contract will be awarded in the second period of the project. |  |
| --- | --- |

### Indirect Cost Rate

- Briefly explain the [indirect cost](https://docs.gatesfoundation.org/documents/indirect_cost_policy.pdf) rate being charged on this project and the rationale and assumptions behind it.

### Currency Exchange

- What is the operating currency you will be using to manage project financials? If different from USD, please explain your organization’s foreign exchange policy, including how and when U.S. Dollars are converted to local currency. (If no formal policy exists, explain the approach you plan to follow on this project.)

The operating currency for this project will be the Nigeria naira (NGN). We plan to use the official exchange rate of the Nigeria central bank and get updated information to guide our judgment from the Nigeria central bank website and Oando.com

- If the project is exposed to additional currencies, other than your operating currency and USD, please name these currencies and provide a rough estimate on how much will be spent in these currencies.

N/A

- Are there any measures you plan to take for reducing currency risk?

N/A

### Other Sources of Support

- Beyond the requested funding from this grant, will the total cost of this project require contributions of funding from other sources (either from your organization or others)?

_No__No (skip questions below)___Yes (please explain)

Please fill out the Total Project Cost and Funding Plan sections in the Excel Budget template and provide further context and explanations here, such as timing, uncertainties/dependencies and important restrictions that apply. If applicable, please also describe any expected in-kind contributions (e.g.,drug donations, personnel time), and explainhow dollarvalues wereestimated.

- If any of the funding (other than this grant) is not yet secured, please describe what you will do if it does not become available.

### Geography Served

- List all countries, sub-regions, and/or states that will benefit from this work and the associated dollar amountsfor the full term of the grant. Please avoid listing "World," unless the work truly serves beneficiaries globally. If geographies include the United States, please indicate city and state. The total of "Foundation Funding" amounts should match therequested grant funding.Add more rows as needed.

For additional information, see the [Geography and Location of Work – Frequently Asked Questions](https://docs.gatesfoundation.org/documents/geography-frequently-asked-questions.pdf).

| Geography Served | Foundation Funding Amount (USD) |
| --- | --- |
| Nigeria | $299,928 |
|  | $ |
|  | $ |

### Location of Work

- List all countries, sub-regions, and/or stateswhere this work would be performed, and associated dollar amountsfor the full term of the grant. This includes your location and any locations where you plan to make payments to individuals or organizations with grant funds. If locations include the United States, please indicate city and state. The total of "Foundation Funding" amounts should match therequested grant funding. Add more rows as needed.

For additional information, see the [Geography and Location of Work – Frequently Asked Questions](https://docs.gatesfoundation.org/documents/geography-frequently-asked-questions.pdf).

| Location of Work | Foundation Funding Amount (USD) |
| --- | --- |
| Nigeria | $299,928 |
|  | $ |
|  | $ |

- Many countries, including India, Vietnam, Bangladesh, and Pakistan require organizations conducting activities funded with foreign funds to comply with local registrations or other requirements. These restrictions may apply to funds you disburse to sub-awardees under this project. Please confirm that your organization will ensure compliance with any such requirements, including taking reasonable steps to ensure that any subgrantees subject to India's Foreign Contribution Regulation Act (FCRA) are in compliance with this law and the regulations thereunder (e.g., depositing subgrant funds only into accounts maintained at the State Bank of India, New Delhi Main Branch and ensuring such subgrantees do not further subgrant funds).

___Not applicable_Confirmed__Confirmed___Not confirmed (please explain)

### Anti-Terrorism and Use of Funds

- Did you list any locationsof work as "to be determined" (TBD) or geographic areas instead of specific countries (e.g., Sub-Saharan Africa or Middle East)?

_No__No___Yes

- Did you list any locations of work that are subject to U.S. government sanctions as identified below? No

| Comprehensive Sanctioned Countries | List-based Sanctioned Countries |
| --- | --- |
| ___Cuba  ___Ukraine’s Crimea Region or so-called Luhansk or Donetsk People’s Republics  ___Iran  ___North Korea  ___Syria | ___Countries subject to list-based sanctions*  **Note: Countries subject to list-based sanctions can be found on the*[*U.S. Department of Treasury website*](https://home.treasury.gov/policy-issues/financial-sanctions/sanctions-programs-and-country-information) |

- If you selected ‘yes’ to TBD locations or locations that are not country-specific, and/or selected locations that are subject to [U.S. government sanctions](https://home.treasury.gov/policy-issues/financial-sanctions/sanctions-programs-and-country-information) or believe that project work could reasonably occur in a location that is subject to U.S. government sanctions, please review the guidance in the [Office of Foreign Assets Control (OFAC) Guidelines](https://docs.gatesfoundation.org/Documents/OFAC_Guidelines_for_External_Use.pdf) and confirm that: (a) you have discussed the location of work with your foundation contact, and (b) your organization will ensure compliance throughout the term of the project with the [anti-terrorism clause](https://docs.gatesfoundation.org/Documents/Sample-Terms-and-Conditions.pdf) in our grant agreement.

___Confirmed	___Not confirmed (please explain)

## Roles and Responsibilities

### Conduct and Control

In answering the questions in this section, please consider all project activities, such as those involving: confidential or protected information (including personally identifiable information or protected health information); the inclusion of children or vulnerable populations; research involving human subjects; clinical trials; post-approval studies; field trials; experimental medicine; provision of medical services (diagnostic, prophylactic or treatment); product development; use of genetically modified organisms, human tissue, animals, radioactive isotopes, pathogenic organisms, recombinant nucleic acids, select agents or toxins, [dual-use technology](https://www.phe.gov/s3/dualuse/Pages/default.aspx), or any substance, organism, or material that is toxic or hazardous; use of aircraft, unmanned vehicle systems, drones or satellites; and the import, export, transfer, approvals, consents, records, data, specimens, images, and materials related to any of the foregoing.

Please confirm that your organization:

- Will maintain the expertise necessary to conduct, control, manage, and monitor all aspects of the project in compliance with all applicable ethical, legal, regulatory, and safety requirements including applicable international, national, state, local, and institutional, school district or school network standards and policies and is responsible for determining and complying with these requirements and standards;
- Will not disclose any confidential or protected information to the foundation without obtaining prior written approval from the foundation and all necessary consents to disclose such information;
- Acknowledges that any activities by the foundation in reviewing documents, providing input or funding does not modify your organization’s responsibility for determining and complying with all applicable ethical, legal, regulatory, and safety requirements for the project in all places;
- Is a government agency, public institution or multilateral organization or will otherwise maintain insurance coverage sufficient to cover the activities, risks, and potential omissions of the project in accordance with generally-accepted standards and as required by law (for instance, general, professional, clinical trial, product liability, medical malpractice, workers' compensation, or otherwise); and
- Will not transfer any biological materials, chemicals, reagents, hazardous materials, or the like to the foundation.

Confirmed___Confirmed___Not confirmed (please explain)

- Does the project involve any of the following: clinical trial, other trial involving human subjects, post-approval study, experimental medicine, field trial of genetically modified organisms, or the provision of medical/health services?

_No__No	___Yes (please explain)

If yes, please list all approvals and consents required for each site and describe the time frame in which your organization will acquire the necessary approvals and consents.

Ethical approval will be obtained from the Federal Ministry of Health of Nigeria, while further approval will be obtained from the Institutional Review Board (IRB) of the Federal University of Technology Owerri, Nigeria. These will be obtained with 4 to 6 weeks of initial application.

- If yes, please identify the name of the entity that will be the sponsor/responsible party of any clinical trials, studies involving human subjects, experimental medicine studies, post-approval studies, products, or regulatory filings contemplated by the project.

*Note: The foundation will not serve as the sponsor/responsible party nor accept delegation of any of these responsibilities. If the project will not involve such activities, please indicate not applicable or N/A below.*

### Advocacy and Lobbying

- While the foundation funds a broad range of advocacy activities, U.S. law prohibits foundation funds from being earmarked to support direct or grassroots lobbying communications. You should answer “Yes” to this question if this project will include engaging with the public or government partners to influence government policy decisions or actions. Does your project include advocacy activities?

___No_Yes__Yes (please explain)

If “yes,” please describe how the project will be conducted to ensure foundation funds are not earmarked to support grassroots or direct lobbying, as summarized in the foundation’s [Advocacy Guidelines](https://docs.gatesfoundation.org/Documents/advocacy-guidelines.pdf), identifying any potential legislative actions related to your advocacy goals. Your response should demonstrate your understanding of the relevant restrictions and exceptions and explain how you will manage compliance during the term of the project. If relevant, also explain how you will comply with other pertinent lobbying rules or requirements (e.g., local, state, or federal disclosure rules).

The partner Non Governmental Organizations (NGOs) involved in this project will be responsible for advocacy and sensitization, awareness reaising and mobiilization of project communities.

# SECTION B – PROGRESS NARRATIVE(S)

**This section is for progress reporting on funded grants.**For each required progress report, complete a version of this progress narrative section and submit the Investment Document to your foundation contact by the date indicated in your grant agreement or latest amendment. If you have any questions or need support, please reach out to your foundation contact. Please find contact information in Section A – Investment Details. The progress narrative is your opportunity to update the foundation on the following:

- Progress made toward achieving your project's stated outputs and outcomes
- Any adjustments to the project
- Financial update for the reporting period
- Feedback for the foundation

Reminder: This document must be submitted in English and as a Word file. PDFs and other file types will not be accepted.

**IMPORTANT:**For each subsequent progress report, do not overwrite existing progress narratives. Instead, copy the following questions and paste to the end of this section before Section C – Final Narrative.

## Progress Reporting Period 1

| Progress Report Number | Reporting Period Start Date | Reporting Period End Date | Progress Report Due Date | Scheduled Payment Amount(if applicable) |
| --- | --- | --- | --- | --- |
|  |  |  |  | $ |

### Progress Details

- What progress have you made toward achieving the project's desired outcomes? Describe what you did in this reporting period and what work is planned or anticipated for the next period.

### Outputs/Funded Developments and Background Technology Update

- Update the table below to reflect completed and/or adjusted outputs, and new outputs/Funded Developments not previously reported. See your original results table in Section A of your proposal for reference. Add more rows as needed.

| Number | Output/Funded Development Description | Newly Added Output/Funded Development? | Status  (e.g., planned, in progress, complete, canceled) | Target  Completion Date* | Actual  Completion Date | Third-Party agreement required? If yes, by when? | Will any IP rights be filed/generated? |
| --- | --- | --- | --- | --- | --- | --- | --- |
| 1 |  | No/Yes |  | [DD Month YYYY] | [DD Month YYYY] | No/Yes | No/Yes |
| 2 |  | No/Yes |  |  |  | No/Yes | No/Yes |
| 3 |  | No/Yes |  |  |  | No/Yes | No/Yes |
| 4 |  | No/Yes |  |  |  | No/Yes | No/Yes |

**Note:For items that are complete or canceled, this should be the date that was stated in the most recent progress report or in the proposal if this is the first report. For items that are not yet complete, either restate the most recent target date or propose a revised target date (e.g.,~~30 Nov 2023~~ 31 Jan 2024).*

- If necessary, please add further explanation below.

- Are there any additional Background Technologies to report which were not described in any documents previously submitted to the foundation?

___No___Yes (please explain)

### Project Adjustments

- What adjustments to the project (scope, timeline, resources) have you made over the past period or are you proposing to make?

### Global Access Update

- Are there any updates to your responses to the Global Access questions in Section A?

___No	___Yes (please explain)

### Roles and Responsibilities Update

- Do you represent that the project activities were conducted in compliance with all applicable ethical, legal, regulatory and safety requirements?

___Yes___No (please explain)

- Are all the project activities described in the documents previously submitted to the foundation?

___Yes___No (please explain and describe any new project activities)

### Financial Update

The purpose of this section is to help the foundation understand expenditures in relation to the work performed under this grant during the most recent reporting period and the upcoming one(s). Please provide actual expenditures for past periods and projected expenditures for future periods by updating the Excel budget template and use this narrative document to share additional explanations. Together, this narrative section and Excel budget should provide a complete quantitative and qualitative description of the current financial state of the project.

For additional instructions, see the [Grant Budgeting & Financial Reporting Detailed Instructions](https://docs.gatesfoundation.org/documents/budget_template_instructions.docx).

#### Latest Period Variance

**“Latest period variance”** compares expenditures that occurred in the most recent reporting period against the projections that were provided at the beginning of the same period. See the “Financial Summary & Reporting” sheet in the Excel budget template for the calculated variance (for example, column AD, starting on row 29 for period 1). The budget template also includes an “Analytics” sheet for convenient calculation of variances.

- Did the project spend more or less than anticipated? At a high level, what are the reasons for spending more or less than projected (such as, programmatic changes, delays in recruitment, etc.)?

- Please provide a detailed explanation for any expense category in which the variance was greater than 10%. This should include an explanation of relevant project decisions or changes to actual costs compared to prior assumptions.

#### Future Period Projections

**In the Excel budget template, please provide estimated expenditures ("projections") for all future periods of the grant (for example, in the** “Financial Summary & Reporting” sheet, after period 1, populate the yellow cells in column T and following). It is important that your projections are realistic and consider the latest plan of activities with updated costs. For example, projections should not just carry forward previously unspent budget amounts into the next period without reassessing expected spending for each future period. In total, the projections should not exceed the total approved grant amount. The budget template’s “Analytics” sheet may be helpful for comparing current to previous projections.

*Note****:*** *Over the course of a grant’s life, the foundation may adjust the timing and amounts disbursed to the grantee. This is done to avoid oversized cash balances on grantee bank accounts when spending is slower than originally budgeted. Realistic and up-to-date projections are a crucial input for right-sizing disbursements.*

- How have your projections for the remaining periods changed compared to your previous projections (from your most recent progress report, or original proposal if this is the first report) and why? Please discuss any project adjustments and shifts in scope, assumptions, timing, decisions etc. which will affect spending. If you had previous discussions with the foundation on changes, please mention.

#### Subawards (if applicable)

If you are an Indian organization/entity subject to India’s Foreign Contribution Regulation Act (FCRA), then in compliance with FCRA, do you confirm that your project does not include any subgrantees? This prohibition does not preclude subcontracts in the ordinary course of business.

___Not applicable     ___Confirmed     ___Not confirmed (please explain)

.

- In the table below, please provide information for all subgrantees or subcontractors receiving grant funds. The total of the column titled *Amount disbursed to subawardee in most recent reporting period* should equal the actual subaward expenses reported for this period in the Excel budget template's “Financial Summary & Reporting” sheet. Add more rows as needed.

| Type of Subaward (grant or contract) | Organization Name | New and/or newly identified subawardee? | Amount disbursed to subawardee in most recent reporting period (USD) | Total amount disbursed to subawardee to date* (USD) | Total amount spent by the subawardee to date* (USD) | Total subaward amount (USD) |
| --- | --- | --- | --- | --- | --- | --- |
|  |  | No/Yes | $ | $ | $ | $ |
|  |  | No/Yes | $ | $ | $ | $ |
|  |  | No/Yes | $ | $ | $ | $ |
| Total | | | **$** | **$** | **$** | **$** |

**As of the end of the most recent reporting period and since the grant start date.*

- If not already addressed in your answers above, please provide additional explanations on subaward spending below. In particular, for subawards that are large or where actual and projected spending are significantly different from prior projections, please explain. *Note: The foundation may request updated budget files for large subawards.*

#### Other Sources of Support and External Factors (if applicable)

- Please provide an update on other sources of support and external factors that affect the financial situation of this project. This may include information on co-funding (updates to the sources listed in the Funding Plan, recent commitments secured, funding gaps and contingency plans). Please also comment on interest earned/spent, and currency exchange impacts (as applicable).

#### Geography Served

- List all countries, sub-regions, and/or states that benefit from this work and the associated dollar amountsfor the full term of the grant. The total of "Foundation Funding" amounts should match the approvedand/or most recently amendedgrant total. If any of the amounts have changed since your last report (or original proposal if no prior report), please mark with an ‘X’ in the third column below. As a reminder, please avoid listing "World," unless the work truly serves beneficiaries globally. If geographies include the United States, indicate city and state. Add more rows as needed.

For additional information, see the[Geography and Location of Work – Frequently Asked Questions](https://docs.gatesfoundation.org/documents/geography-frequently-asked-questions.pdf).

| Geography Served | Foundation Funding Amount (USD) | Has the amount changed since the proposal or last report? (if yes, mark with an ‘X’) |
| --- | --- | --- |
|  | $ |  |
|  | $ |  |
|  | $ |  |

#### Location of Work Updates (if applicable)

- List all locations (countries, sub-regions, and/or states) where this work is being performed andthe associated dollar amountsfor the full term of the grant. The total of "Foundation Funding" amounts should match theapproved and/or most recently amended grant total. If any of the amounts have changed since your last report (or original proposal if no prior report), please mark with an ‘X’ in the third column below. As a reminder, if locations include the United States, please indicate city and state. Add more rows as needed.

For additional information, see the [Geography and Location of Work – Frequently Asked Questions](https://docs.gatesfoundation.org/documents/geography-frequently-asked-questions.pdf).

| Location of Work | Foundation Funding Amount (USD) | Has the amount changed since the proposal or last report? (if yes, mark with an ‘X’) |
| --- | --- | --- |
|  | $ |  |
|  | $ |  |
|  | $ |  |

- If you listed any TBD locations or locations that are not country-specific, and/or locations that are subject to [U.S. government sanctions](https://home.treasury.gov/policy-issues/financial-sanctions/sanctions-programs-and-country-information) or believe that project work could reasonably occur in a location that is subject to U.S. government sanctions, please review the guidance in the [Office of Foreign Assets Control (OFAC) Guidelines](https://docs.gatesfoundation.org/Documents/OFAC_Guidelines_for_External_Use.pdf) and confirm that: (a) you have discussed the location of work with your foundation contact, and (b) your organization will ensure compliance throughout the term of the project with the [anti-terrorism clause](https://docs.gatesfoundation.org/Documents/Sample-Terms-and-Conditions.pdf) in our grant agreement.

___Confirmed___Not confirmed (please explain)

### Feedback for the Foundation

- What are 1–3 ways that the foundation successfully enabled or supported your work during this project? What are 1–3 ways that the foundation can improve?

### Grantee Confirmation

By submitting this report, I declare that I am authorized to certify, on behalf of the grantee identified under Section A – Investment Details, that I have examined the responses provided and related attachments, and that to the best of my knowledge, they are true, correct, and complete.

I also confirm that the grantee identified under Section A – Investment Details has complied with all of the terms and conditions of the grant agreement, including but not limited to the clauses regarding Use of Funds, Anti-Terrorism, Subgrants and Subcontracts, and Regulated Activities.

| Your Name | Report Submitted Date |
| --- | --- |
|  |  |

# SECTION C – FINAL NARRATIVE

**For completed grants only.** At the end of your grant, complete this final narrative section and submit to your foundation contact by the date indicated in your grant agreementor latest amendment. If you have any questions or need support, please reach out to your foundation contact. Please find contact information under Section A – Investment Details.

This is your final update to the foundation, please include:

- The results you achieved for the entire project
- Your perspective on what you learned
- Final financial update
- Feedback you have on the foundation’s support of your work

| Grant Start Date | Grant End Date | Final Report Due Date | Remaining / Unexpended Grant Funds (if applicable) | Remaining / Unexpended Interest (if applicable) | Remaining / Unexpended Currency Gains (if applicable) |
| --- | --- | --- | --- | --- | --- |
|  |  |  | $ | $ | $ |

### Final Results

- Provide information about what you achieved during the grant and your progress towards achieving the project's desired outcomes and outputs.

### Outputs/Funded Developments and Background Technology

- Please update the results table below to reflect completed and/or adjusted outputs, and new outputs/Funded Developmentsnot previously reported. Add more rows as needed.

| **Number** | Output/Funded Development Description | Newly Added Output/Funded Development? | Status  (e.g., planned, in progress, complete, canceled) | Target  Completion Date* | Actual  Completion Date | Third-Party agreement required? If yes, by when? | Were any IP rights be filed/ generated? |
| --- | --- | --- | --- | --- | --- | --- | --- |
| 1 |  | No/Yes |  | [DD Month YYYY] | [DD Month YYYY] | No/Yes | No/Yes |
| 2 |  | No/Yes |  |  |  | No/Yes | No/Yes |
| 3 |  | No/Yes |  |  |  | No/Yes | No/Yes |
| 4 |  | No/Yes |  |  |  | No/Yes | No/Yes |

**Note:For items that are complete or canceled, this should be the date that was stated in the most recent progress report or in the proposal if this is the first report. For items that are not yet complete, either restate the most recent target date or propose a revised target date (e.g.,~~30 Nov 2023~~ 31 Jan 2024).*

- If necessary, please add further explanation below.

- Do you confirm that you have provided a full and accurate disclosure of all outputs/Funded Developments and Background Technology as applicable to the project?

___Yes___No (please explain)

### Global Access

- Do you confirm that you have ensured and will continue to ensure Global Access for the Funded Developments of the project and, as applicable, Background Technology?

___Yes___No (please explain)

### Financial Update

The purpose of this section is to help the foundation understand expenditures in relation to the work performed under this grant during the most recent reporting period. Please provide actual expenditures for past periods by updating the Excel budget template and use this narrative document to share additional explanations. Together, this narrative section and Excel budget should provide a complete quantitative and qualitative description of the financial state of the project.

For additional instructions, see the [Grant Budgeting & Financial Reporting Detailed Instructions](https://docs.gatesfoundation.org/documents/budget_template_instructions.docx).

#### Latest Period Variance

**“Latest period variance”** compares expenditures that occurred in the most recent reporting period against the projections that were provided at the beginning of the same period.

- Did the project spend more or less than anticipated? At a high level, what are the reasons for spending more or less than projected?

- Please provide a detailed explanation for any expense category in which the variance was greater than 10%. This should include an explanation of relevant project decisions or changes to actual costs compared to prior assumptions.

#### Subawards(if applicable)

- If you are an Indian organization/entity subject to India’s Foreign Contribution Regulation Act (FCRA), then in compliance with FCRA, do you confirm that your project did not include any subgrantees? This prohibition does not preclude subcontracts in the ordinary course of business.

___Not applicable     ___Confirmed     ___Not confirmed (please explain)

.

- In the table below, please provide information for all subgrantees or subcontractors receiving grant funds. The total of the column titled *Amount disbursed to subawardee in most recent reporting period* should equal the actual subaward expenses reported for this period in the Excel budget template's “Financial Summary & Reporting” sheet.

| Type of Subaward (grant or contract) | Organization Name | New and/or newly identified subawardee? | Amount disbursed to subawardee in most recent reporting period (USD) | Total amount disbursed to subawardee to date* (USD) | Total amount spent by the subawardee to date* (USD) | Total subaward amount (USD) |
| --- | --- | --- | --- | --- | --- | --- |
|  |  | No/Yes | $ | $ | $ | $ |
|  |  | No/Yes | $ | $ | $ | $ |
|  |  | No/Yes | $ | $ | $ | $ |
| Total | | | **$** | **$** | **$** | **$** |

**Over the life of the grant, from start date to end date*

- If not already addressed in your answers above, please provide additional explanations on subaward spending below. In particular, for subawards that are large or where actual spending was significantly different from prior projections, please explain. *Note: The foundation may request separate updated budget files for sub-awards.*

#### Other Sources of Support and External Factors (if applicable)

- Please provide an update on other sources of support and external factors that affected the financial situation of this project. This may include information on co-funding, interest earned/spent, and currency exchange impacts (as applicable).

#### Geography Served

- List all countries, sub-regions, and/or states that benefit from this work and the associated dollar amountsfor the full term of the grant. The total of "Foundation Funding" amounts should match theapproved and/or most recently amended grant total. If any of the amounts have changed since your last report (or original proposal if no prior report), please mark with an ‘X’ in the third column below. As a reminder, please avoid listing "World," unless the work truly serves beneficiaries globally. If geographies include the United States, indicate city and state. Add more rows as needed.

For additional information, see the[Geography and Location of Work – Frequently Asked Questions](https://docs.gatesfoundation.org/documents/geography-frequently-asked-questions.pdf).

| Geography Served | Foundation Funding Amount (USD) | Has the amount changed since the proposal or last report? (if yes, mark with an ‘X’) |
| --- | --- | --- |
|  | $ |  |
|  | $ |  |
|  | $ |  |

#### Location of Work Updates (if applicable)

- List all locations (countries, sub-regions, and/or states) where this work is being performed and the associated dollar amountsfor the full term of the grant. The total of "Foundation Funding" amounts should match theapproved and/or most recently amended grant total. If any of the amounts have changed since your last report (or original proposal if no prior report), please mark with an ‘X’ in the third column below. As a reminder, if locations include the United States, please indicate city and state. Add more rows as needed.

For additional information, see the [Geography and Location of Work – Frequently Asked Questions](https://docs.gatesfoundation.org/documents/geography-frequently-asked-questions.pdf).

| Location of Work | Foundation Funding Amount (USD) | Has the amount changed since the proposal or last report? (if yes, mark with an ‘X’) |
| --- | --- | --- |
|  | $ |  |
|  | $ |  |
|  | $ |  |

- Do you confirm that you have complied throughout the term of the project with the [anti-terrorism clause](https://docs.gatesfoundation.org/Documents/Sample-Terms-and-Conditions.pdf) in our grant agreement?

___Confirmed	___Not confirmed (please explain)

### Lessons Learned

- What are 1–3 lessons that you learned from this project? If you were to do this project again, how would you approach things differently?

### Feedback for the Foundation

- What are 1–3 ways that the foundation successfully enabled or supported your work during this project? What are 1–3 ways that the foundation can improve?

### Grantee Confirmation

By submitting this report, I declare that I am authorized to certify, on behalf of the grantee identified under Section A – Investment Details, that I have examined the responses provided and related attachments, and that to the best of my knowledge, they are true, correct, and complete.

I also confirm that the grantee identified under Section A – Investment Details has complied with all of the terms and conditions of the grant agreement, including but not limited to the clauses regarding Use of Funds, Anti-Terrorism, Subgrants and Subcontracts, and Regulated Activities.

| Your Name | Report Submitted Date |
| --- | --- |
|  |  |

1. Legal Name will be used in the agreement and should match the name on the bank account that receives the grant funds (assuming fully executed agreement).
2. Trade Name or d/b/a (“doing business as”) only required if different from Legal Name.
3. A fiscal agent is a third-party organization that is not the grantee. The fiscal agent, although not the grantee, signs the grant agreement in addition to the grantee and will receive the grant payments.
