# Supplementary material for: Protocol for the crowdsourced image-based morbidity hotspot surveillance for neglected tropical diseases (CIMS-NTDs)
Source: PLoS One. 2024 May 10;19(5):e0303179. doi: 10.1371/journal.pone.0303179 (PMC11086871; doi:10.1371/journal.pone.0303179)
Supplement: Attachment — Submitted filename: Response to Reviewers.docx [file pone.0303179.s003.docx]

REBUTTAL TO POINTS RAISED BY THE ACADEMIC EDITOR AND THE REVIEWRS

| **Section** | **Point Raised** | **Response** |
| --- | --- | --- |
| Abstract; Introduction | This introduction is quite lengthy. Authors should summarize in max 5 lines.  I would expect that the authors would clearly express that this study aims to develop and test a new methodology | The abstract introduction has been summarized and re-presented in a more succinct manner |
| Abstract; Methods | Authors also need to re-write/re-structure this section; with clear details about the study area, participants, study design/approach, timings, brief details of the newly developed tool, and how it would be used, details on the evaluation protocol; what the expected results would be and the likes. | The methods section of the abstract has been re-presented using the prompts as suggested by the reviewer. |
| Abstract; Ethics and Dissemination | Authors should re-write this as well, 1/2 lines for ethics  and a separate sub-section for funding.  e.g., Funding: This study received financial support from XYZ (Grant number: XYZ) | This sub-section has been re-introduced as Ethical Approval with a stand-alone sub-section for funding. |
| Introduction; line 66 | Not all of them. So kindly rephrase this | Some of the NTDs are marked for elimination, some for control, some for eradication while some for elimination as a public health problem. The statement has be re-written to reflect this. |
| Introduction; line 71 | could these come up before line 66-71 | The paragraph truly sounds like it should introduce the subject matter. It has been brought up to begin the introduction. |
| Introduction; line 85 | It is important to emphasize the challenges that necessitate the prose on traditional surveillance. | The challenges are the problem with the traditional NTDs surveillance method. This has been elaborated and brought up before line 85. |
| Introduction; line 87 | this should as well be referenced | A proper citation has been attached to the statement |
| Introduction; line 89 | this should come up around line 85, and should be referenced | A proper citation has been provided and the statement has been moved to line 85. |
| Introduction; line 102 | this can start as a new paragraph, and of course rephrased:  consider staring like; | The authors agree with this argument. We have moved it to a new paragraph and we have also rephrased the statement. |
| Introduction; line 105 | This could have been the first sub-section under the methodology section.  "Development of XYZ" | This statement is introducing our study objectives as part of our introduction. We believe that that is where it should be. For clarity’s sake, we have rephrased the statement. |
| Introduction; line 106 | This could have been the first sub-section under the methodology section.  "Development of XYZ" | We also believe that this is still part of our introduction which was later elaborated in the methodology. We have also rephrased the statement for better understanding. |
| Introduction; line 111 | this entire section needs to be rephrased and reported in a more succinct style. | We have rephrased and presented in a more succinct style. |
| Introduction; line 115 | If the images were uploaded by third-parties on behalf of affected persons, since most affected persons may live in resource constrained settings, where access to smart-phones, electricity and internet may be lacking. Then one would wonder who gets the feedback proposed here  Then the line 119-121, where the authors mentioned that hygiene practices can also be provided is confusing; authors should be very specific on what would or would not be implemented | Though in our methods section, this objective is elaborated, for clarity, we have added additional statement that shows that the CCPs who obtains and forward NTDs manifestation photographs on behalf of the subject will also be employed to take feedback to the subject. The CCPs will be trained in basic counselling provision for home management of common NTDs. Regarding hygienic practices. The subject will be counselled and trained on basic home care of NTDs like elephantiasis and trachoma that requires regular cleaning with warm water and antiseptic disinfectants. |
| Introduction; line 122 | This section is also not clear enough. What exactly would | The section has been re-written. The objective is the development and AI/ML algorithm model that will help in the prompt and accurate identification and annotation of morbidity images that will be fed into it to specific NTDs, providing a sort of differential diagnosis. Thus, eliminating the need for human annotators as we currently have. |
| Introduction; line 128 | This section should also go into the last paragraph of the discussion | Even though the protocol manuscript has no discussion section. The statement truly seems like a good concluding statement, we have therefore, moved it to the very last paragraph of the manuscript, after the statement of how the research finding will be disseminated. |
| Materials and Methods; line 142 | this entire section should serve as the justification for developing the novel methods and should fit into the introductory section (the last paragraphs that leads to the aims/objective of the study) | We are not too sure that the PLOS ONE protocol manuscript for publication should contain a justification section; in the interim, we have created a justification sub-section under the introduction and we have moved the statement therein because we agree that the statement is actually justifying the need for the study. |
| Materials and Methods; line 176 | Needs to be referenced | Appropriate reference has been provided. |
| Materials and Methods; line 187 | Please rephrase this | The statement has been re-presented in a clearer form. |
| Materials and Methods; line 190 | this is the first mention of this acronym | NGOs like WHO, UNICEF etc are common acronyms that often time do not require interpretation in reports. The acronym has however, been written in full. |
| Materials and Methods; line 192 | The study design is still not clear. Author should used a flow chat to describe the implementation process; in the intervention and cotrol states, who would be the target audience, and how will the interventions be deployed. | Kindly refer to the “Description of Processes” section in line 198 which has now been re-named as “Study Design”. Also refer to the project’s comprehensive flowchart (Fig 1) in Appendix |
| Materials and Methods; line 198 | This should be merged with the study design section which I suggested above | A study design section has been created and this has been made a sub-section under the newly created study design section |
| Materials and Methods; line 306 | Throughout this process, it is still not clear how the NTDs photographs would be sourced/obtained from the community | The process of photograph collection was described in line 208 – 245 where we described our advertisement materials. We then give the description of our study sites in line 247 – 270. We explained that advert notice will be put out in our study sites calling for NTDs subjects and their community members to send us photos of NTDs manifestations upon notice through our dedicated WhatsApp and Telegram channels. We also described the process of community entry, engagement and sensitization and the selection of CCPs within the people that will help us to get manifestation photographs of subjects who cannot send us photographs on their own. |
| Materials and Methods; line 308 | this section should be described separately; the first section on how to manage the photographs received; and the second section on the AI development etc | This activity will commence in the 4th month of our project inception. It will continue simultaneously with other activities including morbidity images crowdsourcing from the communities. We are presenting it here because the entire process was described within their timelines of implementation. |
| Materials and Methods; line 327 | this should be discussed simultaneously with other section, and not separately as described here. | The section has been cut out and joined positioned directly under the section that describes the Intervention communities' mobilization and sensitization |
| Data Management Plane; line 384 | This is the first time of mentioning this, I think the detailed methodology and platform should have been described earlier before getting to this stage | This is not the first time we mentioned WhatsApp and Telegram in the protocol, it was first mentioned in the Abstract, then under study aim that has been changed to Justification then it was mentioned under “description of process” |
| Data Management Plane; line 391 | Authors could merge this section with the Outcome Measure section, and title the section as "Study Indicators". Under this section they should describe the data, and also the explanatory and outcome variable, and also the planned statistical analysis | They have been merged. |
| Ethical considerations and informed consent; line 432 | Please delete this, there is no reason to have the protocol number in this section | Protocol number has been removed from the statement. |
| Discussion; line 455 | this should be specified; what demographic data; sex, age, name or what | The types of demographic data that will be obtained has been clearly spelt out. |
| Discussion; line 457 | on the long run, would this be achieved? when providing care to this population, the healthcare worker would still be aware. Are there reports of stigmatization from HCW to patients or among community members and those affected | Note that the care that each subject will receive based on their specific diagnosis will be personalized and likely to be delivered to in the homes of these subjects. The stigmatization here in most cases does not emanate from the HCW but from other members of the community. The CIMS-NTDs method prevent the situation where a subject is made to wait endlessly at the health center trying to access care. |
| Discussion; line 466 | what measures of | This comment is not clear. The acronym there is CCP (Community Contact Person) and not CPP. |
| Discussion; line 478 | to | Correction taken |
| **ACADEMIC REVIEWERS COMMENT** | | |
| Comment 1 | Please ensure that your manuscript meets PLOS ONE's style requirements, including those for file naming | The manuscript has been re-designed to meet the PLOS ONE’s style |
| Comment 2 | In your Methods section, please include additional information about your dataset and ensure that you have included a statement specifying whether the collection and analysis method complied with the terms and conditions for the source of the data. | The Methods section has been enhanced to include additional information about our dataset. Our data collection and analysis method will be in conformity with the terms and conditions for source data. We have included a statement indicating this under our ethical consideration and informed consent section |
| Comment 3 | Thank you for stating the following in the Acknowledgments Section of your manuscript: "We would like to express our heartfelt gratitude to the following organizations and entities for their invaluable support and contributions to this research project; The Bill and Melinda Gates Foundation for their generous funding, which made this study possible. The Nigeria Federal Ministry of Health NTD Programme Office for their collaboration and partnership throughout the project.  Please remove any funding-related text from the manuscript | The funding-related texts has been removed from the manuscript |
| Comment 4 | In your Data Availability statement, you have not specified where the minimal data set underlying the results described in your manuscript can be found. PLOS defines a study's minimal data set as the underlying data used to reach the conclusions drawn in the manuscript and any additional data required to replicate the reported study findings in their entirety. All PLOS journals require that the minimal data set be made fully available. | This is a proposal of an ongoing project; we are yet to generate or analyse any data that may yield a valid conclusion. No result was described in the proposal manuscript. |
|  |  |  |
|  |  |  |
|  |  |  |
|  |  |  |
