# Supplementary material for: Protocol for the crowdsourced image-based morbidity hotspot surveillance for neglected tropical diseases (CIMS-NTDs)
Source: PLoS One. 2024 May 10;19(5):e0303179. doi: 10.1371/journal.pone.0303179 (PMC11086871; doi:10.1371/journal.pone.0303179)
Supplement: Attachment — Submitted filename: Second Responses to Reviewers.docx [file pone.0303179.s004.docx]

**RESPONSES TO POINT RAISED BY THE REVIEWERS**

| **Section** | **Point Raised** | **Response** |
| --- | --- | --- |
| General | This revised version requires language editing | The manuscript has been subjected to English language editing, it was improved for grammar, spellings, punctuations and clarity. |
| General | As the previous review comments were suggestions to improve the study, the authors have selected what feels right for them. The writing of this protocol flows better in the arranged format. | Our review was made in the light of the recommendations from the academic editors and the two peer reviewers. We have made effort to made the manuscript as smooth flowing as possible through further English language editing which was done by an English expert. |
| References | References are not sequential in the Introduction section, in Line 62 you started with 1,3,4 and only in line 78 do you cite reference 2. | It was an oversight from the editing author, the error has been corrected. The references are now properly presented in the order of appearances. |
| Introduction | Social media handles mentioned in line 107, the rest of the protocol is mentioning WhatsApp and Telegram. Does this mean other social media will be considered in future? Mention that in line 107. | We made use of the phrase social media handles to give a blanket name to WhatsApp and Telegram platform. These are the most common platforms that the Nigerian population are conversant with. We do not plan to include other social media handles in the future, we have therefore replaced the phrase “social media handles” with “WhatsApp and Telegram” |
| Materials and methods;  Study setting | The Pilot sites are 6/36 of states in Nigeria, include this information in the demographics paragraph to give the reader a clear perspective of the size of the country. Are all 36 states NTDs endemic? | All the 36 states in Nigeria plus the FCT have at least one NTD being endemic.  A statement mentioning the number of states in Nigeria and the number of the pilot study sites have been included in the “Study setting” sub-section. |
| Description of process | It is not clear how this data is going to be managed, there is something about IP, but I think some explanation is required to clarify this point | Activities in the Reception, collation, processing and analysis of photo and demographic data, and other data (Month 4-15) starting from line 308 – 330 described the manners with which the received data will be managed.  The use of IP number- internet protocol number of the device that will send the data to us is just to add another layer of security to the data management activities. The IP number of the devices that sends in photo-data will be identified to allow the researchers to know where the data is coming from, this will help us checkmate the situation of multiple submission of the same data thereby maintain the integrity of the submitted data.  In addition to this, the section on “Data Management and Analysis Plans” also gave a succinct description of how data that will be received will be dealt with prior to forwarding to our experts for annotation and what will be done with the annotated image-data. |
| Comment by Reviewer 2 | | |
|  | I believe the authors have satisfactorily responded to all the comments raised earlier on. However, they have introduced a new section on Justification. my initial suggestion to them was to just include a few lines to justify the protocol, as part of their introduction. I guess this must have been wrongly understood. Authors should try to reconcile, and the lines in the new justification section are kind of repetitive of those earlier raised in the introduction. | The JUSTIFICATION sub-heading has been collapsed into the INTRODUCTION section with the two merged together while avoiding duplication of points raised. |
